# A model for reticular dysgenesis shows impaired sensory organ development and hair cell regeneration linked to cellular stress

**DOI:** 10.1242/dmm.040170

**Published:** 2019-12-20

**Authors:** Alberto Rissone, Erin Jimenez, Kevin Bishop, Blake Carrington, Claire Slevin, Stephen M. Wincovitch, Raman Sood, Fabio Candotti, Shawn M. Burgess

**Affiliations:** 1 Translational and Functional Genomics Branch, National Human Genome Research Institute (NHGRI), National Institutes of Health (NIH), Bethesda, MD, USA; 2 NHGRI Zebrafish Core, Translational and Functional Genomics Branch, NHGRI, NIH, Bethesda, MD, USA; 3 NHGRI Cytogenetics and Microscopy Core, NHGRI, NIH, Bethesda, MD, USA; 4 Division of Immunology and Allergy, Lausanne University Hospital and University of Lausanne, Lausanne, Switzerland

**Keywords:** Ak2, Reticular dysgenesis, SCID, Hearing loss, Zebrafish, Hair cells, Lateral line, Antioxidants

## Abstract

Mutations in the gene *AK2* are responsible for reticular dysgenesis (RD), a rare and severe form of primary immunodeficiency in children. RD patients have a severely shortened life expectancy and without treatment die, generally from sepsis soon after birth. The only available therapeutic option for RD is hematopoietic stem cell transplantation (HSCT). To gain insight into the pathophysiology of RD, we previously created zebrafish models for Ak2 deficiencies. One of the clinical features of RD is hearing loss, but its pathophysiology and causes have not been determined. In adult mammals, sensory hair cells of the inner ear do not regenerate; however, their regeneration has been observed in several non-mammalian vertebrates, including zebrafish. Therefore, we used our RD zebrafish models to determine whether Ak2 deficiency affects sensory organ development and/or hair cell regeneration. Our studies indicated that Ak2 is required for the correct development, survival and regeneration of sensory hair cells. Interestingly, Ak2 deficiency induces the expression of several oxidative stress markers and it triggers an increased level of cell death in the hair cells. Finally, we show that glutathione treatment can partially rescue hair cell development in the sensory organs in our RD models, pointing to the potential use of antioxidants as a therapeutic treatment supplementing HSCT to prevent or ameliorate sensorineural hearing deficits in RD patients.

## Abstract (precis)

**Summary:** A zebrafish model of reticular dysgenesis reveals hair cell developmental deficits that can be partially rescued by antioxidants, pointing to their potential use as a therapeutic treatment for reticular dysgenesis patients.

## INTRODUCTION

Reticular dysgenesis (RD) is a rare form of severe combined immunodeficiency (SCID), a heterogenous group of immunological diseases usually characterized by profound defects to the T and B lymphoid lineages (Lagresle-Peyrou et al., 2009; Pannicke et al., 2009). In particular, RD patients also have a reduced number of neutrophils, with a specific block at the promyelocyte stage, and an unresponsiveness to granulocyte colony stimulating factor stimulation in combination with severe sensorineural hearing loss (Aloj et al., 2012; Hoenig et al., 2017). The immunodeficiencies associated with strong neutropenia leads these patients to recurrent severe infections and premature death within the first weeks of life. For these patients, a hematopoietic stem cell transplantation (HSCT) represents the only available treatment, but, because of myeloid-lineage defects, myeloablative conditioning is necessary in order to increase the chances of full engraftment of the donor stem cells (Hoenig et al., 2018). However, the HSCT does not improve the non-hematological defects, such as the hearing loss, which has a huge impact on the cognitive functioning and quality of life of these children (Eggermont, 2017; Mittal et al., 2017).

Mutations in the adenylate kinase 2 (*AK2*) gene have been shown to be responsible for the disease (Lagresle-Peyrou et al., 2009; Pannicke et al., 2009; Rissone et al., 2015). AK2 is an enzyme localized to the intermembrane space of the mitochondria, where it plays an important role in sustaining mitochondrial respiration and cellular energy metabolism (Dzeja and Terzic, 2003; Oshima et al., 2018; Six et al., 2015). Because the knockout of mouse *Ak2* showed an early embryonic lethality (Kim et al., 2014; Rissone et al., 2015), other cellular and animal models needed to be developed. Insect models of AK2 deficiency indicated an essential role of the gene in embryonic growth and cell survival (Chen et al., 2012; Horiguchi et al., 2014). They also suggested that maternal *ak2* mRNA can, at least initially, compensate for the lack of *ak2* gene zygotic transcription. In zebrafish, AK2 knockdown induced by morpholino injection showed hematopoietic defects without affecting general embryonic development (Pannicke et al., 2009; Rissone et al., 2015). These results were confirmed by two different mutant alleles carrying frameshift mutations in zebrafish *ak2* exon 1 and a missense mutation in exon 4 (Rissone et al., 2015). Similar to what was observed *in vitro* in patient fibroblasts and CD34^+^ bone marrow cells (Pannicke et al., 2009; Six et al., 2015), zebrafish mutants presented an increased level of cellular oxidative stress leading to apoptosis and cell death (Rissone et al., 2015). Notably, these phenotypes can be reduced by the administration of antioxidants in both zebrafish mutants and, moreover, the same kind of treatment was able to rescue myeloid differentiation *in vitro* in induced pluripotent stem cells (iPSCs) obtained from fibroblasts of an RD patient (Rissone et al., 2015).

Although most of the work linked AK2 function to its bio-energetic activity, other evidence highlighted the presence of alternative roles, partially unrelated to its enzymatic activity and/or the mitochondrial localization (Hoenig et al., 2018). The AK2 protein associates with dual-specificity phosphatase 26 (DUSP26), resulting in the suppression of cell proliferation by FADD dephosphorylation (Kim et al., 2014). In addition, AK2 is involved in an amplification loop that ensures the execution of intrinsic apoptosis via an interaction with FADD and caspase 10 (Lee et al., 2007). Previous reports showed that AK2 deficiency impairs the regular induction of the unfolded protein response (UPR) mechanism *in vitro* (Burkart et al., 2011; Tanimura et al., 2014). Finally, using RD patient-derived iPSCs, recent work showed a reduction of nuclear ATP levels in AK2-deficient cells during specific stages of hematopoietic differentiation (Oshima et al., 2018). Reduced levels of nuclear ATP could be responsible for the altered transcriptional profile observed during hematopoietic differentiation (Oshima et al., 2018; Six et al., 2015). Overall, these lines of evidence suggest that, at least to some extent, the cellular AK2 roles can be cell-type or context specific.

Sensorineural hearing loss is the most common form of human hearing loss and it can be due to several different factors including genetic mutations, ototoxic compound exposure, aging, infectious diseases or environmental stress, such as prolonged exposure to excessive noise (Eggermont, 2017; Kindt and Sheets, 2018). In general, all these different factors can induce damage to the mechanosensory hair cells in the organ of Corti or the stria vascularis and they can also impair the function of the spiral ganglion neurons or of the more proximal auditory structures (Cunningham and Tucci, 2017). Because of the limited regenerative ability of mammals, hair cells cannot regenerate after damage and the resultant hearing loss is permanent. In contrast, non-mammalian vertebrates like zebrafish possess a huge regenerative potential and they can replenish hair cells during homeostasis or after damage, providing a model in which to study hair cells development and, in particular, hearing restoration after damage (Kniss et al., 2016; Lush and Piotrowski, 2014b; Whitfield, 2002).

Although zebrafish inner ears lack a structure strictly equivalent to the mammalian cochlea, most of the functions present in all other vertebrates (i.e. hearing and vestibular functions) are fully conserved (Whitfield, 2002). Moreover, zebrafish possess another mechanosensory system on their body surface called the lateral line, which provides feedback information about water flow along the body of the fish (Ghysen and Dambly-Chaudiere, 2007; Nuñez et al., 2009; Pujol-Martí and López-Schier, 2013). The lateral line is composed of a network of several small sense organs, the neuromasts, distributed over the zebrafish body in a specific pattern (Ghysen and Dambly-Chaudiere, 2007). This sensory system comprises two major branches, an anterior part that extends on the head (anterior lateral line or ALL), and a posterior part on the trunk and tail (posterior lateral line or PLL). Each neuromast consists of a central core of hair cells that are innervated by lateralis afferent neurons. The hair cells are then surrounded by two different types of non-sensory cells: supporting and mantle cells (Ghysen and Dambly-Chaudiere, 2007). Because of its structural simplicity and experimental accessibility, the lateral line, and in particular the PLL, has become a very popular model in which to study hair cell development and regeneration and it has contributed significantly to the understanding of the molecular pathways that control those phenomena (Monroe et al., 2015).

To shed light on the causes of RD hearing loss, we used zebrafish models of RD (Rissone et al., 2015). Our data showed that zebrafish *ak2* is expressed in inner ear and neuromast structures and its deficiency severely affects the survival of hair cells in those structures through an increased level of oxidative stress and cell death. Finally, the administration of glutathione (GSH) to zebrafish *ak2* mutants showed that antioxidant treatments were able to partially increase the number of hair cells in the inner ear and PLL of *ak2*-deficient animals and that this increase was at the level of both development and long-term survival of the hair cells.

## RESULTS

### The zebrafish *adenylate kinase 2* gene is expressed in sensory organs

**Fig. 1.**
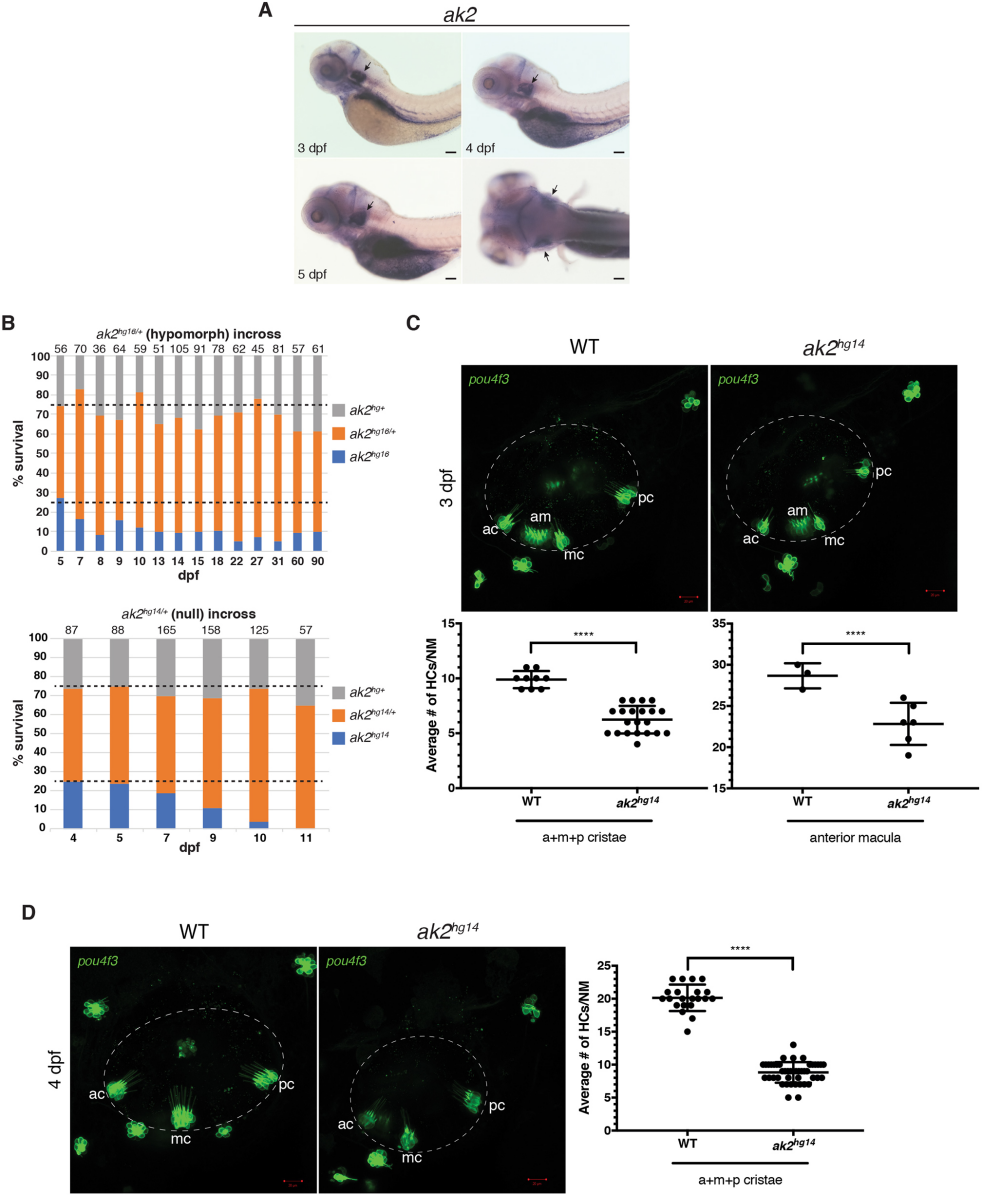
***ak2* larval expression in the otic vesicle and sensory phenotypes of the zebrafish *ak2* alleles.** (A) WISH on wild-type zebrafish larvae at different developmental stages. Lateral (3-5 dpf) and dorsal (5 dpf) views. Black arrows point to *ak2* expression in the otic vesicles. Scale bars: 200 μm. (B) Survival rates for wild-type, *ak2* heterozygous, and *ak2* mutant larvae at different dpf. Top and bottom graphs show survival of the progeny from an incross of *ak2^hg16/+^* or *ak2^hg14/+^* heterozygous animals, respectively. Dashed lines indicate the expected ratio following Mendelian inheritance. Numbers above the bars indicate the total number of embryos used for genotyping analysis at each stage. (C,D) Comparison of confocal maximum projections of inner ear regions (dashed circles) from Tg(*pou4f3*:GAP-GFP) wild type (WT) and *ak2^hg14^* mutants at 3 (C) and 4 (D) dpf. Scale bars: 20 µm. For each set of images, graphs show the comparison of the average number of GFP^+^ hair cells in the anterior macula (3 dpf) and cristae (3 and 4 dpf) between *ak2*-deficient animals and controls (WT). a, anterior; ac, anterior crista; am, anterior macula; HCs, hair cells; m, medial; mc, medial crista; NM, neuromast; p, posterior; pc, posterior crista. The error bars indicate s.d. ****P<0.0001 (two-tailed unpaired Student's test).

Reticular dysgenesis is a rare hematological disorder caused by mutations in the adenylate kinase 2 (*AK2*) gene (Lagresle-Peyrou et al., 2009; Pannicke et al., 2009). Although at least one RD patient with skeletal defects has been reported so far (Al-Zahrani et al., 2013), the only non-hematological clinical feature required for the diagnosis of RD is sensorineural deafness/hearing disability (Hoenig et al., 2017, 2018). Because of the paucity of data, overall the pathophysiology of the hearing loss in RD patients is essentially uncharacterized. Here, we used our zebrafish models of RD to understand better the physiopathology of hearing loss in RD patients. Initially, we tested whether the zebrafish AK2 gene (*ak2*) was expressed in sensory organs such as the inner ear or the lateral line system. Whole-mount *in situ* hybridization (WISH) analysis of endogenous *ak2* expression during zebrafish development showed that from 3 days post-fertilization (dpf) *ak2* is expressed in different anatomical regions, including strongly in the otic vesicle (Fig. 1A, Fig. S1B). Transversal sectioning of 5 dpf stained embryos showed that *ak2*-expressing cells were present in different structures of the inner ear such as the cristae and the anterior and posterior maculae (Fig. S1A). Although we were not able to observe *ak2* expression in neuromasts in whole-mount staining, probably due to low levels of expression, in the same transverse sections we also found positive cells inside the neuromasts of the ALL (Fig. S1A, black arrowheads). Based on their location, rounded morphology and large nuclei (Sahly et al., 1999), those cells represent putative hair cells. Moreover, independent single cell RNA-seq analysis performed on neuromast cells from 5 dpf transgenic zebrafish (Lush et al., 2019) confirmed *ak2* expression in hair and supporting cells (Fig. S1B).

These data suggest that, in zebrafish, *ak2* is expressed in sensory organs and that the lack of its expression could potentially affect inner ear or neuromast development.

### Lack of *ak2* affects inner ear hair cell development

In a previous study, we showed that two different *ak2* mutants, carrying a missense mutation (previously denoted as *ak2^L124P/L124P^*, and here indicated as *ak2^hg16^*) or frame-shift mutations in exon1 of *ak2* (*ak2^del2/del2^* and *ak2^ins4/ins4^* mutants, here designated as *ak2^hg14^* and *ak2^hg15^*, respectively), presented very similar severe hematopoietic phenotypes affecting hematopoietic stem and precursor cell development (Rissone et al., 2015). However, survival analysis showed that a reduced percentage of *ak2^hg16^* mutants can reach adulthood (∼10% compared with the expected percentage of ∼25%) (Fig. 1B, top panel). In contrast, *ak2*-deficient mutants *ak2^hg14^* and *ak2^hg15^*, lacking the *ak2* mRNAs (Rissone et al., 2015), start to die around 5 dpf; and by 11 dpf we were not able to find any surviving homozygous mutants (Fig. 1B, bottom panel; data not shown). These data suggest that, despite the severity of the hematopoietic defects observed in all *ak2* mutants, *ak2^hg16^* likely represents a hypomorphic mutation, whereas the other two alleles (*ak2^hg14^* and *ak2^hg15^*) are more likely fully null. Notably, in *ak2^hg16^* adults we did not observe any behavioral signs indicating potential sensorineural defects (Nicolson et al., 1998; Whitfield, 2002) such as ‘circling’ or the lack of a startle response. When we compared hair cell bundle density of inner ear structures from *ak2^hg16^* and their heterozygous siblings (*ak2^hg16/+^*) of the same age, we did not find a statistically significant difference (Fig. S2).

Notably, using the Tg(*pou4f3*:GAP-GFP) transgenic line to visualize the mature hair cells in null mutants, we found a statistically significant difference in the average number of hair cells in 3 and 4 dpf developing inner ears of *ak2^hg14^* mutants compared with their control siblings (Fig. 1C,D).

Taken together, these data suggest that a more severe *ak2* deficiency is needed to impair the correct development of the hair cells in the zebrafish inner ears compared with the hematopoietic defects seen in the hypomorphic allele.

### *ak2* deficiency affects PLL development

**Fig. 2.**
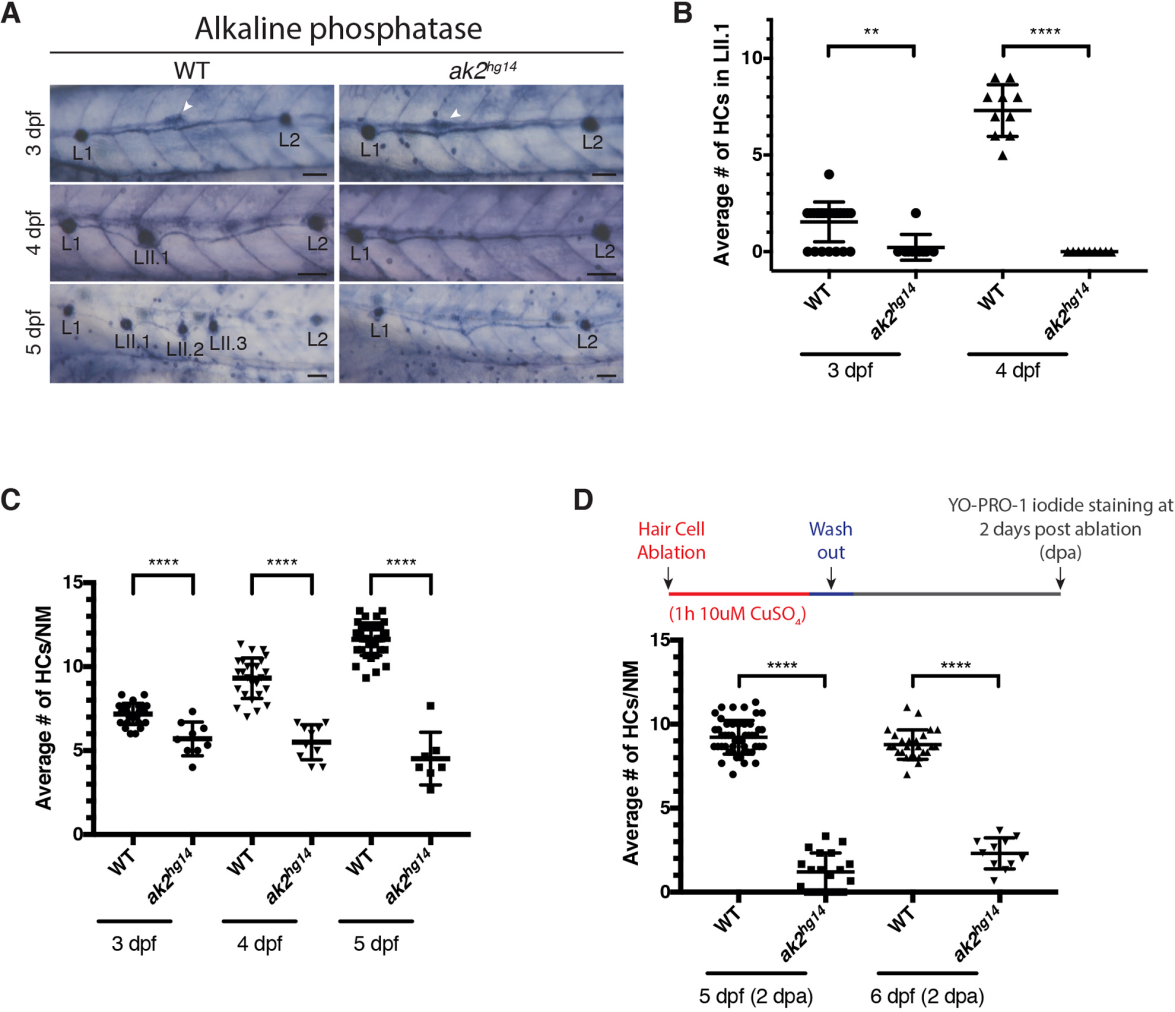
**Reduced hair cell number and secondary neuromast differentiation phenotypes in *ak2^hg14^* mutants.** (A) Alkaline phosphatase staining of 3-5 dpf *ak2^hg14^* homozygous embryos and their siblings (WT). White arrowheads indicate the migrating secondary primordium (primII). L1-2 and LII.1-3 designate primary and secondary neuromasts, respectively. Scale bars: 50 μm. (B,C) Yo-PRO-1 iodide staining of hair cells (HCs) in lateral line neuromasts in *ak2^hg14^* embryos and their wild-type siblings during development to compare the average number of HCs in LII.1 secondary neuromasts (B) and in primary neuromasts (C). (D) Average number of HCs per primary neuromast in Yo-PRO-1 iodide-stained *ak2^hg14^* embryos and their siblings 2 days after ablation with CuSO_4_. Mean±s.d.**P<0.0011; ****P<0.0001 (unpaired, two-tailed Student's *t*-test).

One hypothesis for the sensorineural hearing loss in AK2-deficient patients is that the composition of the luminal space is compromised, resulting in hair cell loss (Lagresle-Peyrou et al., 2009; Hoenig et al., 2018). To understand whether hair cells in a different context (i.e. not in a luminal space) could similarly be affected by the lack of *ak2* expression, we examined the PLL of our knockout fish. In zebrafish larvae, the PLL possesses at least three different populations of neuromasts denoted as primary, secondary and intercalary (IC) (Nuñez et al., 2009). Primary neuromasts are deposited by the first migrating primordium (primI), which travels from the otic vesicle along the midline of the trunk on both sides of the embryo (Chitnis et al., 2012; Ghysen and Dambly-Chaudiere, 2007; Ma and Raible, 2009) to the tail, regularly depositing primordial neuromasts. Moreover, all the primary neuromasts are connected by a thin continuous stripe of cells (interneuromast cells or INCs), which, later during development, act as multipotent stem cells to produce IC neuromasts (Thomas et al., 2015). By 2 dpf, when the migration of the first primordium is complete as it reaches the most caudal part of the trunk of the embryos, a second, slower primordium (primII) starts its migration from the otic vesicle following a similar route to that used by the primI and it generates a second set of neuromasts called ‘secondary neuromasts’ (or LII) (Nuñez et al., 2009). In *ak2^hg16^* hypomorphic embryos, we did not observe any major defects to the PLL formation as indicated by alkaline phosphatase (AP) staining (Venero Galanternik et al., 2016) at 4-5 dpf or Yo-PRO-1 staining (Behra et al., 2009; Santos et al., 2006) of functional hair cells from 3 to 5 dpf (Fig. S3A,B). Additionally, we did not observe any statistical difference in the regenerative ability of hair cells after copper sulfate ablation (Hernández et al., 2006; Mackenzie and Raible, 2012) (Fig. S3C). In contrast, from ∼3 dpf *ak2^hg14^* embryos presented a lack of positive AP signal (Fig. 2A) and Yo-PRO-1 staining (Fig. 2B) in secondary neuromasts (LII.1-3), suggesting late defects to secondary neuromast formation. Notably, although the overall development of primary neuromasts appeared normal in *ak2^hg14^* embryos (Fig. 2A), further analysis showed a reduction in the average number of Yo-PRO-1^+^ hair cells/neuromast after 3 dpf (Fig. 2C), indicating significant defects during primary neuromast development and maturation. To determine if loss of *ak2* function has a negative impact on the regeneration of hair cells, we checked whether *ak2* expression was present after hair cell ablation with copper sulfate. WISH analysis performed at different time points after complete hair cell ablation with a 1-h treatment with 10 µM copper sulfate, showed that *ak2* expression was increased in neuromasts 3 h post-ablation (hpa) (Fig. S1D). Accordingly, we also observed a strong reduction of Yo-PRO-1^+^ hair cells in *ak2^hg14^* embryos at 2 days post-ablation (dpa) compared with control siblings (Fig. 2D), suggesting there is either no significant capacity for regeneration or, developmentally, the total hair cell number is limited to a lower number of cells with the loss of AK2 activity.

Overall, these data suggest that lack of *ak2* impairs the correct development and maturation of the secondary neuromasts, partially affecting the development and the regenerative ability of the hair cells of the primary neuromasts as well, leading us to hypothesize that hair cell and neuromast precursor cells are being prematurely depleted in *ak2*-deficient animals.

### *ak2* deficiency affects correct expression of neuromast markers in secondary neuromasts

**Fig. 3.**
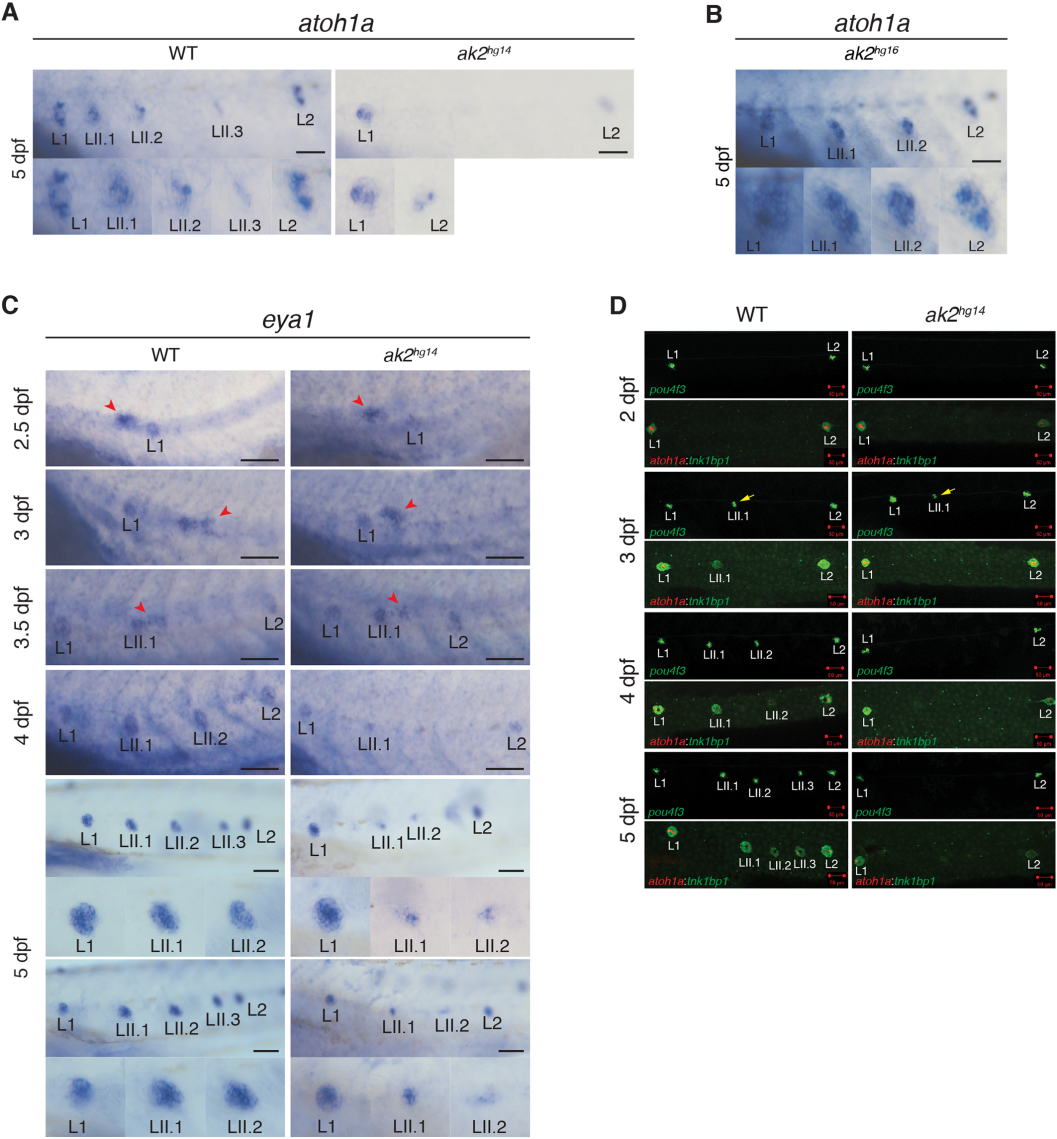
**Characterization of PLL neuromast markers in lateral line of *ak2^hg14^* mutants.** (A,B) WISH analysis of *atoh1a* marker expression in primary and secondary neuromasts on 5 dpf *ak2^hg14^* (A) and *ak2^hg16^* (B) embryos and their siblings (WT). (C) *eya1* expression from 2.5 to 5 dpf in *ak2^hg14^* embryos and their siblings. Red arrowheads indicate the primII. Each panel shows higher magnification of the regions in dashed rectangles from corresponding pictures in Fig. S5. Scale bars: 100 µm. (D) Confocal analysis from 2 to 5 dpf of trunk regions of *ak2^hg14^* embryos and their siblings with different transgenic backgrounds to visualize components of the lateral line rosettes. Scale bars: 50 µm. Yellow arrows indicate *pou4f3*:GFP^+^ HCs in secondary neuromasts.

To characterize further the PLL phenotypes observed in *ak2*-deficient mutants, we performed WISH and confocal microscope analyses of *ak2*-deficient (*ak2^hg14^* and *ak2^hg15^*) embryos in wild-type and different transgenic backgrounds. WISH analysis with the immature hair cell marker *atoh1a* showed a specific reduction of expression in primary neuromasts (L1 and L2) and a complete lack of expression in secondary neuromasts of *ak2^hg14^* embryos at 5 dpf (Fig. 3A, Fig. S4A) that was not observed in control siblings or in the *ak2^hg16^* hypomorphic mutants (Fig. 3B). Interestingly, WISH analysis with the *eya1* marker labeling primII and lateral line neuromasts (Sahly et al., 1999) showed no noticeable defects until 3.5 dpf (Fig. 3C, Fig. S4B). However, from ∼4 dpf *eya1* expression was undetectable in the secondary primordium and strongly reduced or completely absent in LII.1 and LII.2 neuromasts (Fig. 3C, Fig. S4B).

For a more detailed study of the secondary neuromast formation and development in *ak2*-deficient embryos, we crossed *ak2^hg14/+^* and *ak2^hg15/+^* animals with transgenic lines marking different cellular components of neuromasts. As previously observed using Yo-PRO-1 staining (Fig. 2B), the analysis of *ak2^hg14^* Tg(*pou4f3*:GAP-GFP) embryos further confirmed the lack of GFP^+^ hair cells in secondary neuromasts from ∼4 dpf (Fig. 3D). Notably, at 3 dpf the confocal analysis showed the presence of GFP^+^ cells in LII.1 neuromasts, although these were severely reduced in number compared with the controls (Fig. 3D, yellow arrows). The lack of Yo-PRO-1^+^ cells in LII.1 of *ak2* homozygous mutants at the same stage (Fig. 2B) suggested that these GFP^+^ hair cells are not fully mature and functional, despite expressing *pou4f3*.

Using the double-transgenic line Tg(*tnks1bp1*:EGFP; *atoh1a*:dTOM) (Behra et al., 2012) to label immature hair cells (Fig. 3D, red) and supporting cells (Fig. 3D, green), we were able to highlight further defects in secondary neuromast formation. In particular, the analysis showed that *ak2*-deficient embryos presented a specific lack of both dTOM and EGFP signals in LII.1 neuromasts from ∼3 dpf onwards. This is similar to the depletion of hematopoietic stem and precursors cell observed for the zebrafish *ak2* alleles (Rissone et al., 2015).

These data indicate that *ak2*-deficient embryos present an altered expression of markers for different neuromast cell populations, with a reduction in both immature and mature hair cell- and supporting cell-specific markers. However, the persistence in WISH analysis of a faint signal for the *eya1* marker in secondary neuromasts at 5 dpf suggests possible residual traces of secondary neuromasts in that region.

### *ak2* deficiency affects the ventral migration of secondary neuromasts and the development of interneuromast cells

**Fig. 4.**
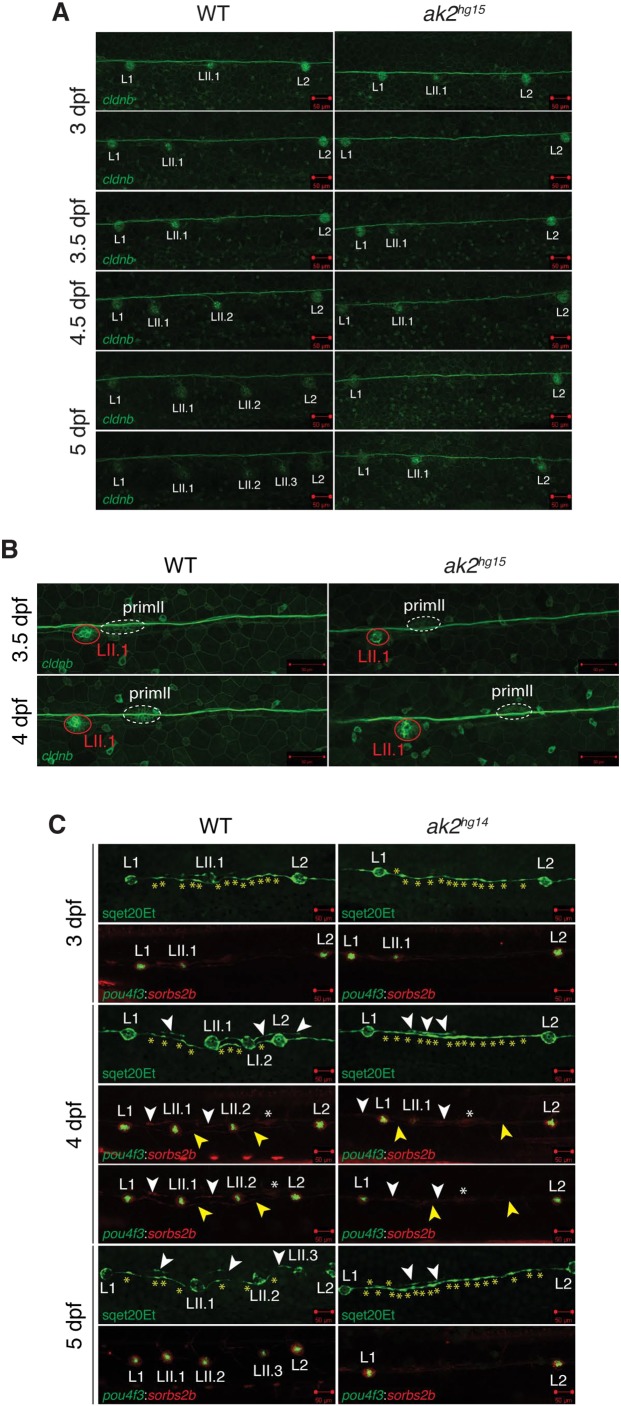
**Analysis of lateral line phenotypes using different *ak2* mutant transgenic lines.** (A) Confocal analysis at different developmental stages of the trunk regions of *ak2^hg15^* embryos and their control siblings (WT) in the Tg(*-8.0cldnb*:LY-EGFP) background. (B) Confocal analysis from 3.5 to 4 dpf of trunk regions of an ak2*^hg15^* embryo and a control embryo in the Tg(-*8.0cldnb*:LY-EGFP) background labeling the migrating secondary primordium (dashed ellipse), deposited secondary neuromast (red circle), connecting interneuromast cells and epithelial cells. Confocal images were collected before and right after the end of the time-lapse confocal movie recording. (C) Confocal analysis from 3 to 5 dpf of trunk regions of *ak2^hg14^* embryos and their siblings in different lateral line transgenic backgrounds. Yellow asterisks label the interneuromast cells between L1 and L2 in the sqet20Et transgenic line. White and yellow arrowheads denote primI or primII interneuromast cells, respectively. White asterisks label the migrating secondary primordium. Scale bars: 50 µm.

To verify the presence of residual secondary neuromasts between L1 and L2, we crossed *ak2^hg15/+^* animals with the transgenic reporter line Tg(-8.0*cldnb*:LY-EGFP). Until 4 dpf, this transgenic line expresses the EGFP reporter in all the different components of the PLL (Haas and Gilmour, 2006). From ∼4 dpf, EGFP expression in the PLL becomes specifically limited to the supporting cells in all neuromasts, the interneuromast cells and the afferent lateral line neurons innervating each neuromast (López-Schier et al., 2004; Lush and Piotrowski, 2014a). Confocal analysis of *ak2^hg15^* embryos and their siblings from 3 to 5 dpf showed that at 3 dpf some of the *ak2*-deficient embryos completely lacked EGFP signal in the LII.1 secondary neuromasts (Fig. 4A). However, in the remaining *ak2^hg15^* population where the LII.1 retained some EGFP-positive signal, secondary neuromast development was severely affected. In particular, we never observed the formation of EGFP^+^ LII.2 neuromasts. In addition, in contrast to wild-type or carrier siblings, the secondary neuromasts remained close to the horizontal myoseptum, at the same level of primary neuromasts (Fig. 4A). Similar migratory defects in secondary neuromasts have only been previously described in the *strauss* mutants (López-Schier et al., 2004). Notably, at 5 dpf there is still a mixture of phenotypes among the *ak2^hg15^* embryos, some of which are totally negative for EGFP^+^ LII.1 neuromasts, whereas others show the presence of the first secondary neuromast, in the absence of further structures. To confirm that the observed phenotypes were not due to early defects of the secondary neuromasts or the inability of primII to migrate, we also performed confocal analysis on *ak2^hg15^* Tg(-8.0*cldnb*:LY-EGFP) mutants and their siblings from 3.5 to 4 dpf (Movies 1 and 2, respectively and Fig. 4B). As summarized in Fig. 4B, we did not observe major differences during primII migration between ∼3 and 4 dpf.

As previously mentioned, IC neuromasts are derived from the proliferation and differentiation of the INCs, a population of precursor cells connecting the neuromasts of the lateral line that was deposited by the primI during its migration (Sapede et al., 2002). In physiological conditions, this population of cells remains quiescent because of the presence of inhibitory signals from glial cells (Grant et al., 2005; López-Schier and Hudspeth, 2005), and isolated IC neuromasts are only rarely observed between L1 and L2 primary neuromasts in wild-type embryos (Lush and Piotrowski, 2014a; Nuñez et al., 2009). However, it has been shown that the lack of inhibitory signals induced by loss of glial cells and/or the PLL nerve (Grant et al., 2005; López-Schier and Hudspeth, 2005) can prompt ectopic production of IC neuromasts (Lush and Piotrowski, 2014a) in the region. Moreover, a previous study showed that ablation of the secondary primordium primII induces ectopic production of IC neuromasts in the same region (Nuñez et al., 2009). Notably, in *ak2*-deficient embryos we never observed sporadic IC neuromasts or an increased number of IC neuromasts between L1 and L2 neuromasts (Figs 2A, 3A-D and 4A), suggesting that the PLL glial and nerve cells are still present and secreting the inhibitory factors. However, the observed phenotypes could be caused by *ak2* deficiency disrupting the formation of the interneuromast cells and IC neuromasts. Alternatively, it is possible that the secondary neuromasts could still be present but lacking in the expression of the tested markers. To investigate these different possibilities, we performed confocal analysis from 3 to 5 dpf on two different transgenic lines in the *ak2^hg14/+^* background: the ET20 line labels mantle cells and interneuromast cells (Parinov et al., 2004), and the double transgenic line Tg(*pou4f3*:GAP-GFP;GBT0002^mn0002GT^) expresses GFP in hair cells and mRFP1 protein tagging the *sorbs2b* gene in supporting cells (Clark et al., 2012; Ding et al., 2016) (Fig. 4C). As previously observed with other markers, the analysis showed an absence of mantle cells in the LII.1 secondary neuromasts, without apparent defects in the mantle cells of the primary neuromasts. We did occasionally observe interneuromast cells derived from primII migration (Fig. 4C, white arrowheads) in *ak2^hg14^* embryos. Notably, from 4 dpf we detected an increased number of interneuromast cells between the L1 and L2 neuromasts (Fig. 4C, yellow asterisks). In the *ak2^hg14^* mutants, primI- and primII-derived interneuromast cells and the primII primordium (yellow and white arrowheads and white asterisks, respectively) were mRFP1^+^ until 4 dpf. Moreover, only some of the null embryos had LII.1 neuromasts positive, at very low levels, for expression of hair and supporting cell markers (Fig. 4C). Finally, at 5 dpf, *ak2*-deficient embryos lacked expression of both GFP and mRFP1 reporters between L1 and L2 primary neuromasts (Fig. 4C) suggesting short-term viability of such cells.

Overall, these data suggest a possible defect in the activation of interneuromast cells with a resulting deficiency in the formation of IC neuromasts. The mRFP1 expression in PLL secondary structures confirmed the presence of LII.1 secondary neuromasts in some *ak2*-deficient embryos at least until 4 dpf. These data are consistent with a premature depletion of the stem cell pool for the lateral line organs.

### Partial impairment of Wnt/β-catenin in *ak2*-deficient mutants

**Fig. 5.**
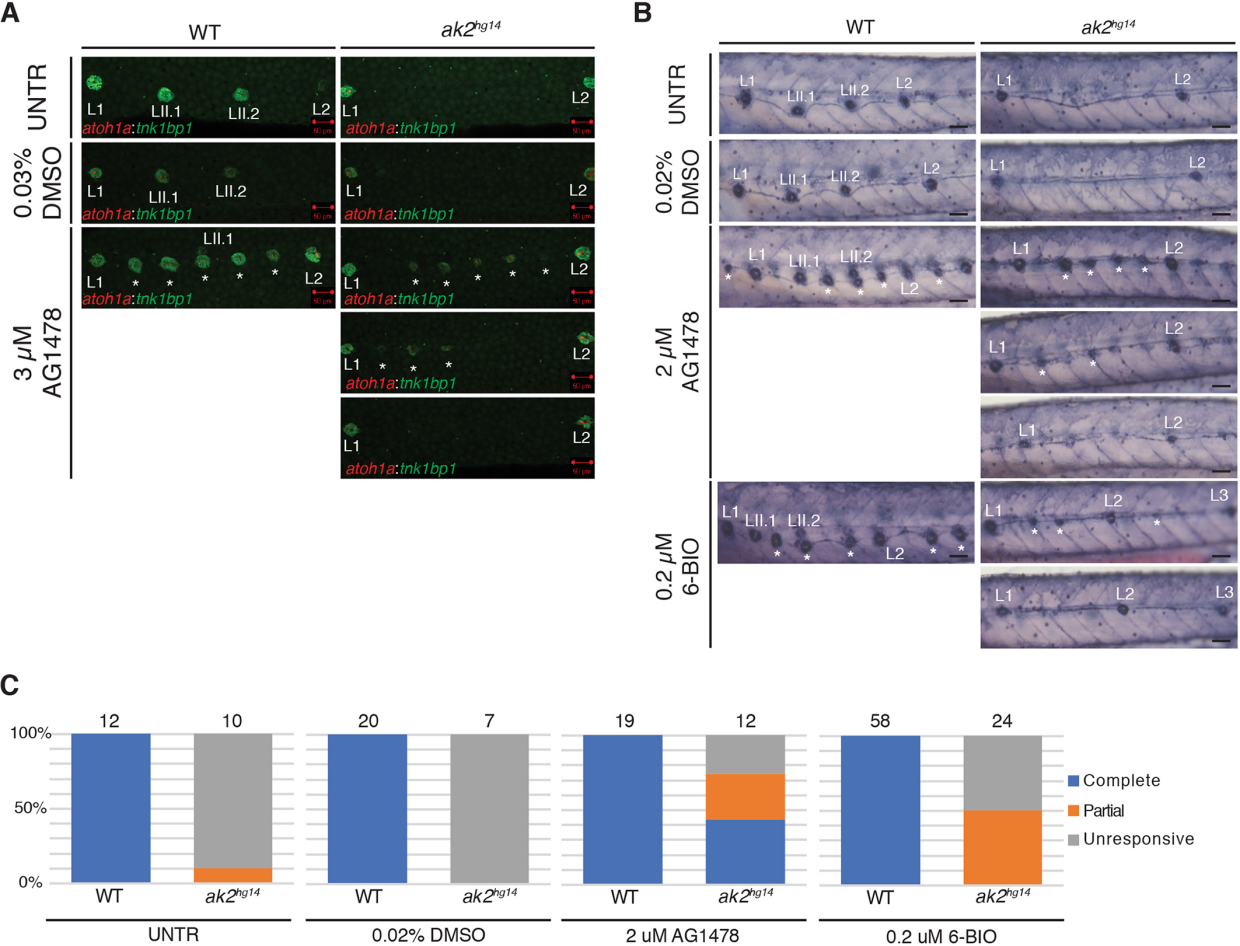
***ak2* deficiency impairs IC neuromast formation.** (A,B) Chemical treatments of *ak2^hg14^* mutants and their control siblings (WT) with AG1478 or 6-BIO. (A) Confocal analysis at 5 dpf of trunk regions for untreated, 0.03% DMSO or 3 µM AG1478-treated embryos in the Tg(*tnks1bp1*:EGFP; *atoh1a*:dTOM) transgenic background. Scale bars: 50 µm. (B) Alkaline phosphatase staining of 5 dpf *ak2^hg14^* and control embryos untreated or treated with 0.02% DMSO, 2 µM AG1478 or 0.2 µM 6-BIO compounds. White asterisks indicate IC neuromasts. Scale bars: 50 μm. (C) Quantification of the effect of AG1478 or 6-BIO treatments in inducing the formation of ectopic ICs in *ak2^hg14^* homozygous mutants and their control siblings. The total number of embryos analyzed per genotype is indicated above each bar.

To verify that in *ak2*-deficient mutants the Schwann cells of PLL did not possess any severe deficiencies, we tested the effects of pharmacological inhibition of ErbB signaling using the tyrosine kinase inhibitor |AG1478 (Osherov and Levitzki, 1994) on *ak2^hg14^* mutant and control embryos in the double-transgenic background Tg(*tnks1bp1*:EGFP; *atoh1a*:dTOM). In particular, we focused our attention on the temporal window from 48 to 50 hours post-fertilization (hpf), which corresponds to the end of the migration of the Schwann cells (Lush and Piotrowski, 2014a). Untreated and DMSO-treated *ak2^hg14^* embryos never produced *atoh1a*:dTOM^+^ and *tnks1bp1*:EGFP^+^ IC neuromasts (Figs 3D and 5A). As was previously reported (Lush and Piotrowski, 2014a), inhibition of ErbB signaling by AG1478 at 50 hpf was sufficient to induce ectopic formation of IC neuromasts in all the control embryos (Fig. 5A). However, in *ak2^hg14^* mutants the treatment showed a partial penetrance, with three major classes of embryos: (1) totally unresponsive (bottom-right panel), (2) partially responsive (middle-right panel) and (3) embryos possessing a number of ectopic neuromasts similar to controls (top-right panel). Because at the tested dose we always observed a uniform response in the control population, the partial penetrance observed in the null mutants seems to reflect a real heterogeneity of the *ak2^hg14^* population's response to the treatment and not just variation in inhibition levels. It is important to note that the induced neuromasts (Fig. 5A, white asterisks) were *atoh1a*:dTOM and *tnks1bp1*:EGFP double positive, although their size was strongly reduced compared with control siblings. Because we never observed the expression of these two markers in the secondary neuromasts of *ak2* mutants, their presence in AG1478-induced mutant neuromasts suggests a difference in the potency of secondary and IC neuromast precursors, probably due to their separate cellular origins.

Although little is known about the signaling pathways that orchestrate IC neuromast formation, a previous study showed that it requires both Wnt/β-catenin and Fgf signaling pathways (Lush and Piotrowski, 2014a). In particular, Wnt/β-catenin signaling is required for initial interneuromast proliferation and Fgf signaling is crucial for subsequent organization of interneuromast cells into a rosette structure and for triggering cellular differentiation (Lush and Piotrowski, 2014a). To determine whether the Wnt pathway was affected in *ak2* mutants, we treated the embryos from an *ak2^hg14/+^* incross with 6-BIO, a potent inhibitor of the GSK-3 enzyme (Fig. 5B). To obtain a quantitative analysis, we performed AP staining of the embryos and we included AG1478 treatment as a further internal control. 6-BIO has been shown to result in strong activation of the Wnt/β-catenin pathway (Meijer et al., 2003) and the formation of extra IC neuromasts in the PLL of treated zebrafish embryos (Lush and Piotrowski, 2014a). As shown in Fig. 5B, in *ak2^hg14^* untreated or DMSO-treated embryos secondary or IC neuromasts between L1 and L2 were always AP negative. At the same time, all of the AG1478 and 6-BIO-treated control embryos developed extra IC neuromasts (Fig. 5B, white asterisks). It's important to note that, in order to avoid 6-BIO toxicity from 24 h of administration, we used it at a concentration of 0.2 µM (which is 10 times lower than the previously published dose; Lush and Piotrowski, 2014a; Nishiya et al., 2014). After 3 days of treatment with the selected dose, we observed a strong induction of ectopic neuromasts in the trunk regions of control embryos, without any major morphological defects or reduced survival of the embryos. These results were very similar to those we obtained on control embryos with a 2 µM AG1418 treatment (Fig. 5B). As previously observed (Fig. 5A), the *ak2*-deficient embryos treated with the AG1478 compound could be classified in three different groups (Fig. 5B,C). AG1478 administration induced the formation of ectopic IC neuromasts in ∼42% (5/12), a partial response in ∼33% (4/12), and a lack of IC neuromast formation in the remaining *ak2*-deficient population (25%, 3/12). Similarly, we found a partial penetrance using the GSK-3 inhibitor 6-BIO, as well. However, in this case we observed that half of the *ak2*-deficient population (12/24) was totally unresponsive to the treatment, whereas the other half showed a very mild response, with a reduced number of ectopic IC neuromasts compared with controls. In both the treatments we noticed that, again, the ectopic IC neuromasts were significantly reduced in size (Fig. 5A,B).

Overall, these data suggest an impairment of Wnt/β-catenin signaling pathways in the interneuromast cells of *ak2*-deficient embryos. The increased proliferation observed in the interneuromast cells of *ak2* null mutants suggests that, in basal conditions, the interneuromast cells seem to be able to respond initially to the Wnt/β-catenin pathway. However, without an external stimulus (like AG1478 or 6-BIO treatments, which induce a sustained Wnt/β-catenin activation), interneuromast cells fail to generate ectopic IC neuromasts.

### *ak2* deficiency induces increased cell death in inner ear, primII and PLL neuromasts

Previous studies have shown that, in RD patient fibroblasts and zebrafish hematopoietic tissues, Ak2 protein has anti-apoptotic functions (Pannicke et al., 2009; Rissone et al., 2015). Therefore, we wondered whether the same phenomena could be the cause of the decreased number of hair cells observed in *ak2*-deficient animals.

**Fig. 6.**
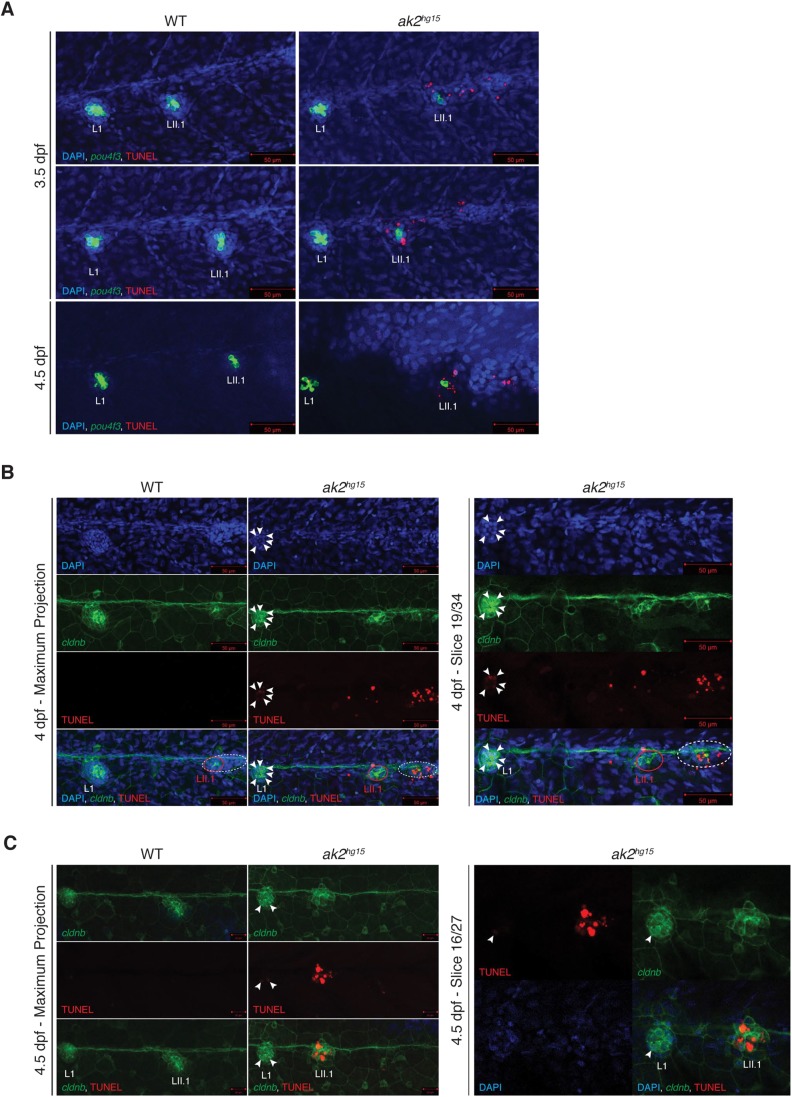
**Increased cell death in the otic vesicles and the PLL of *ak2^hg15^* embryos.** (A) Maximum projections of 3.5 and 4.5 dpf ak2*^hg15^* embryos and their control siblings in the Tg(*pou4f3*:GAP-GFP) background (indicated as *pou4f3*) stained with TUNEL assays (red signal). (B,C) Maximum projections (left panels) and representative single plane confocal analysis (right panels) at 4 (B) and 4.5 (C) dpf of a TUNEL assay (red signal) performed on *ak2^hg15^* embryos and their siblings in the Tg(-*8.0cldnb*:LY-EGFP) background (*cldnb*, green) labeling the migrating secondary primordium (dashed ellipse), deposited primary and secondary (red circle) neuromasts and epithelial cells. White arrowheads label TUNEL-positive cells in the L1 primary neuromast. Nuclei are labeled with DAPI (blue). Scale bars: 50 µm (A,B); 20 µm (C).

To investigate the possible presence of dying hair cells in the inner ear and the PLL structures, we performed terminal deoxynucleotidyl transferase dUTP nick end labeling (TUNEL) assays on *ak2*-deficient embryos and their siblings in the *pou4f3*:GAP-GFP or *cldnB*:LY-EGFP transgenic backgrounds, respectively. Because the organs of interest were on the larval skin, we had to use an optimized TUNEL protocol with a very mild digestion of the embryos (see Materials and Methods for further details). In general, TUNEL staining from 2 to 4 dpf showed an increased level of apoptotic cells in *ak2^hg15^* embryos compared with the controls (red signal in Fig. 6, Fig. S5; data not shown). Analysis of the TUNEL signal distribution in the area surrounding the inner ear in each section of the whole *z*-stack allowed us to verify that, in most cases, the TUNEL positivity corresponded to the nuclei of mature *pou4f3*:GAP-GFP^+^ cells in the neuromasts of the ALL and in the inner ear structures (Fig. S5, yellow arrowheads).

Notably, for the same samples we also observed a strong signal in the trunk regions between L1 and L2 primary neuromasts, suggesting the presence of apoptotic cells in structures that could represent the primII and the secondary neuromasts (Fig. 6A). To verify this possibility, we repeated the analysis on 4 dpf trunk regions of *ak2* mutants in the *cldnb*:LY-EGFP transgenic background. As shown in Fig. 6B, the analysis confirmed the presence of a striking number of TUNEL^+^ cells in the migrating primII, with very few dead cells in the secondary neuromast LII.1. At the same stage, we observed the presence of several TUNEL^+^ cells in L1 primary neuromasts (Fig. 6B, white arrowheads), providing an explanation for the reduced number of hair cells (in combination with a regeneration deficiency) in primary neuromasts previously observed using Yo-PRO-1 staining (Fig. 2C,D). We obtained similar results in LII.1 secondary neuromasts at 4.5 dpf (Fig. 6C), which correlated with the strong reduction of *eya1* observed at similar stages (Fig. 3C).

Therefore, it appears that the *ak2* deficiency induced increased levels of cell death in zebrafish mechanosensory organs combined with an inability to regenerate properly, explaining the reduced number of mature hair cells in the inner ear and the developmental and regenerative defects observed in primary and secondary neuromasts.

### *ak2* deficiency triggers oxidative stress response genes

**Fig. 7.**
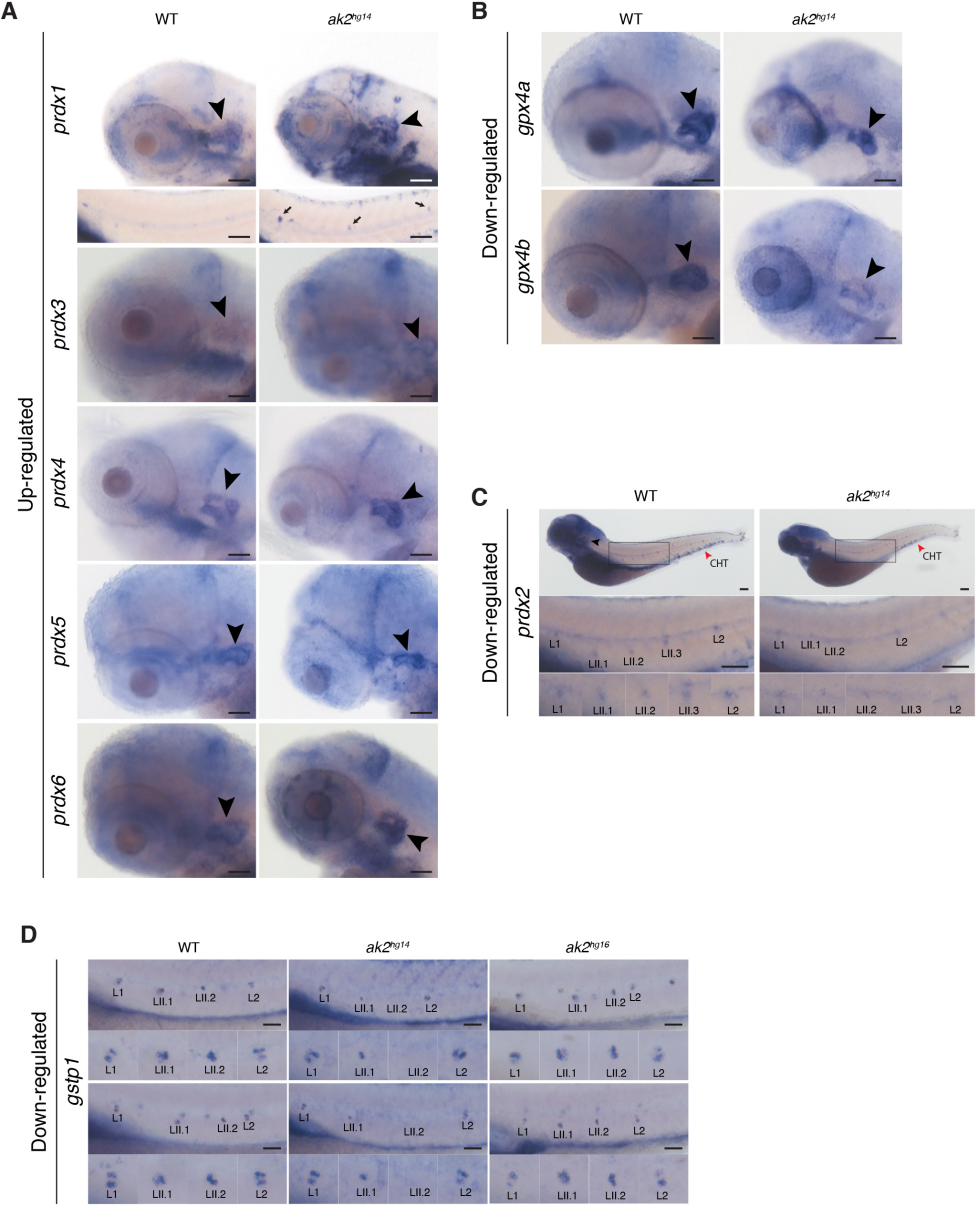
**Altered expression of oxidative stress markers in *ak2^hg14^* embryos.** Expression analysis of oxidative stress markers in *ak2^hg14^* and control sibling (WT) embryos at 4 or 5 dpf. (A) Upregulated expression of several *prdx* genes in the otic vesicle and PLL at 4 dpf. Bottom panel for *prdx1* marker: the black arrows indicate ectopic expression of the marker in PLL neuromasts of the corresponding embryos. (B) Downregulated expression of *gpx4a* and *gpx4b* markers at 4 dpf. (C) Specific downregulation of *prdx2* marker at 5 dpf in PLL neuromasts and caudal hematopoietic tissue (red arrowhead, CHT). Scale bars: 200 µm. (D) Comparison of *gstp1* expression in the PLL neuromasts in control embryos, *ak2^hg14^* null and *ak2^hg16^* hypomorphic mutants at 4 dpf. Two different embryos per genotype are shown. Lateral views with anterior to the left. Black arrowheads indicate the otic vesicle. Scale bars: 100 µm.

Ak2 deficiency has been linked to an increased level of intracellular ROS and oxidative stress (Pannicke et al., 2009; Rissone et al., 2015). In particular, in the hematopoietic tissues of the zebrafish *ak2* models of RD we previously described the induction of heme oxygenase 1a (*hmox1a*) expression (*Rissone et al., 2015*), which represented a defense mechanism for cells against potential damage caused by increased oxidative stress (Loboda et al., 2016; Poss and Tonegawa, 1997). In order to characterize the possible effects of increased levels of oxidative stress induced by *ak2* deficiency in sensory organs, we tested the expression of several genes involved in cellular defense against ROS, including some specific *nrf2* target genes (Ma, 2013; Mukaigasa et al., 2012; Nakajima et al., 2011) (Fig. 7, Fig. S6). Some of these markers were expressed at detectable levels in the inner ears (*prdx4-6*, *gpx1a-b* and *gpx4a-b*) and PLL neuromasts (*prdx2* and *gstp1*) under normal physiological conditions, indicating that they normally participate in basal antioxidant defense in these structures.

Contrary to what was originally predicted based on the presumed high levels of oxidative stress in *ak2* deficiency, we did not observe a uniform increase of expression across the whole set of the markers in *ak2* mutant embryos. Based on how the stress genes responded (up, down or unchanged expression) in the inner ear or the PLL, we divided the genes into three categories (Fig. 7, Fig. S6). Fig. 7A shows that most of the peroxiredoxin (*prdx*) genes showed an increased level of expression in the inner ear. Notably, *prdx1* expression is also upregulated in several other tissues of the body, including the PLL (Fig. 7A; data not shown). Among the *prdx* gene family, the only exception was *prdx2*, which was not expressed in the inner ear and expression of which was reduced in caudal hematopoietic tissue (CHT) and in the primary and secondary neuromasts of the PLL (Fig. 7C). Other examples of genes that were downregulated in *ak2*-deficient embryos were the glutathione peroxidase 4 genes (*gpx4a* and *gpx4b*); in particular, *gpx4b* expression appeared to be strongly reduced in the inner ears (Fig. 7B). The expression of glutathione peroxidase 1a and 1b (*gpx1a* and *gpx1b*) and superoxide dismutase 1 and 2 (*sod1* and *sod2*) appeared to be unchanged between *ak2*-deficient embryos and their control siblings (Fig. S6A,B).

Surprisingly, at 4 dpf the pattern of expression of glutathione S-transferase pi 1 (*gstp1*) seemed to differ significantly between the primary and secondary neuromasts (Fig. 7D). In the secondary LII.2 neuromasts, which are deposited later than LII.1 neuromasts, *gstp1^+^* cells were typically limited to the central part of the neuromast (Fig. 7D). Older primary neuromasts like L1 and L2 presented a specific and repeatable pattern of expression, with *gstp1^+^* cells mostly limited to the dorso-ventral regions of the neuromast, usually with an asymmetrical level of expression, and very low expression in the central part of the more mature neuromasts (Fig. 7D). Secondary LII.1 appeared to represent an intermediate state between the older neuromasts and LII.2, with a broader number of *gstp1^+^* cells and with one region, in this case on the antero-posterior axis, showing higher expression of the marker. This difference in the axis orientation correlated with the different hair cell polarity between primary and secondary neuromasts, which possess an antero-posterior or dorso-ventral orientation, respectively (López-Schier et al., 2004; Nuñez et al., 2009). The variation in the *gstp1* expression pattern seemed to correlate with the different maturation state of each neuromast, consistent with the *gstp1* expression data at earlier stages (2-3 dpf) shown in Fig. S6C and those publicly available through The Zebrafish Information Network (https://zfin.org/) indicating a clear expression of the gene in the most central part of the primary neuromasts (Howe et al., 2013; see also ZFIN Direct Data Submission by Thisse et al., https://zfin.org/ZDB-GENE-020806-4). Moreover, an independent scRNA-seq analysis on 5 dpf neuromasts (Lush et al., 2019) indicates an enrichment of *gstp1* expression in particular in hair cells (Fig. S7).

Compared with control siblings or *ak2^hg16^* hypomorphic mutants, *ak2*-deficient embryos completely lacked *gstp1* expression in the LII.2 neuromast, whereas in LII.1 neuromasts its expression was reduced and restricted only to the most central cells of the neuromast. These data were supported by our previous observations using Yo-PRO-1 staining and confocal analysis (Figs 2B, 3D and 4C), suggesting that *ak2*-deficiency impairs the maturation of LII.1 neuromasts.

Taken as a whole, these results confirm that *ak2* deficiency perturbs the physiological oxidative state of the cells inducing an altered expression of some but not all oxidative stress-related genes. The altered expression of zebrafish NRF2 targets such as *prdx1* and *gstp1* were not uniformly increased suggesting that the specific cellular context could alter the responses of different stress markers and potentially could explain how different tissues displayed different pathologies.

### Antioxidant treatment partially rescued inner ear hair cell and PLL neuromast defects

**Fig. 8.**
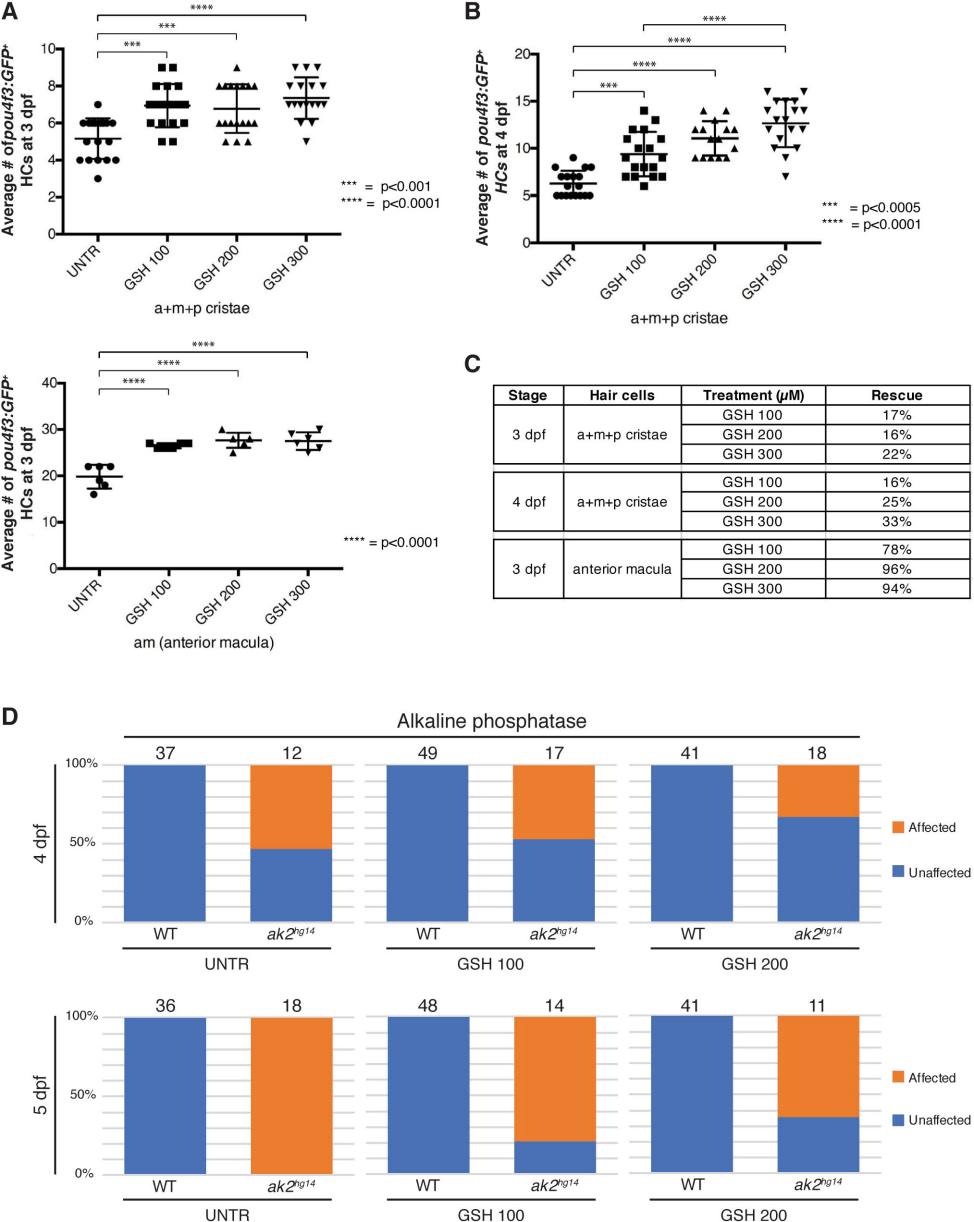
**Antioxidant treatment partially rescues hair cell numbers in the inner ear and the lateral line of *ak2^hg14^* mutants.** (A,B) Average number of mature hair cells in the inner ear cristae (top) and anterior macula (bottom) of 3 dpf (A) or in the inner ear cristae of 4 dpf (B) *ak2^hg14^* embryos treated with different concentrations of GSH (100-300 µM). Results are shown as mean±s.e.m. (one-way ANOVA, followed by Tukey's post hoc test; *P*<0.001). (C) Summary of the percentage of rescue induced by the different GSH treatments on the inner ear cristae and anterior macula at 3 and 4 dpf. (D) Quantitative analysis of the rescue induced by GSH treatments on the expression of alkaline phosphatase in PLL neuromasts of 4 and 5 dpf *ak2^hg14^* and control embryos. Numbers above bars indicate the total amount of embryos used for the analysis. a, anterior; m, medial; p, posterior; UNTR, untreated. A one-way ANOVA (followed by Tukey's post hoc test; *P*<0.001) was used to compare groups of fish treated differently.

Previously, we showed that antioxidant treatment was able to partially rescue the hematopoietic phenotypes observed in zebrafish models of RD (Rissone et al., 2015). In particular, treatment with 10-50 µM GSH reduced the ectopic expression of the oxidative stress marker *hmox1a* and also restored the expression of *c-myb* and *rag1* in the hematopoietic tissues of *ak2*-deficient embryos (Rissone et al., 2015). In addition, the treatment of RD patient iPSCs with GSH was able to increase the *in vitro* differentiation rate of neutrophils (Rissone et al., 2015). We therefore tested if a similar treatment would be beneficial to the sensorineural defects of a zebrafish RD model. We treated the *ak2^hg14^* and control embryos with a range of GSH concentrations. Using the *pou4f3*:GAP-GFP transgenic background, we initially compared the number of mature hair cells in the inner ear cristae and the anterior macula of 3 dpf *ak2^hg14^* and control embryos, untreated or treated with 100-300 µM GSH (Fig. 8A, Fig. S8); then we extended the analysis to the cristae of 4 dpf *ak2^hg14^* and control embryos in the same conditions (Fig. 8B, Fig. S9). Comparison of the number of hair cells in control and mutant animals in different conditions (untreated versus different doses of the treatment) allowed us to calculate the percentage of rescued hair cells induced by the treatments (Fig. 8C). At all of the tested doses the treatment significantly affected the number of hair cells of control embryos (Figs S8 and S9). Notably, the specific comparison between untreated and GSH-treated *ak2^hg14^* mutants showed a statistically significant increase in the average number of hair cells in the cristae of 3 dpf null mutants, with 17%, 16% and 22% of rescued hair cells, respectively (Fig. 8A, top panel, Fig. S8 and Fig. 3C). Similar results were also obtained in the cristae of 4 dpf *ak2^hg14^* and control embryos, where we observed a dose-dependent rescue that was statistically significant; the antioxidant treatments were able to rescue the 16%, 25% and 33% of the hair cells, respectively (Fig. 8B, Figs S8 and S9). Finally, analysis of the average number of hair cells in the 3 dpf macula of *ak2^hg14^* mutants and control siblings showed that the macula seems to be less sensitive to the *ak2* deficiency, with an average of ∼20 hair cells (∼28 in the controls), corresponding to a 29% of reduction of hair cells, compared with the 48% reduction observed in the cristae of untreated embryos at the same stage or the 69% reduction at 4 dpf. Notably, the GSH treatments were able to almost fully restore the physiological number of hair cells in this specific structure with a 78%, 96% and 94% rescue for GSH100, GSH200 and GSH300, respectively. This indicates that the antioxidant agent might be more effective during the first phases of the *ak2* deficiency, suggesting that they might work best when the damage at the target tissue is not yet severe. Unfortunately, because of the anatomical localization of the macula and the consequent difficulties in obtaining good imaging of the structure, we were not able to repeat the analysis at 4 dpf or later, to see if the strong recovery observed at 3 dpf was sustained over time.

We also tested the ability of GSH treatment to rescue secondary neuromast development. As previously shown, from ∼4 dpf secondary neuromasts in *ak2^hg14^* mutants were *atoh1a*, *atoh1a*:dTOM, *tnks1bp1*:EGFP and *pou4f3*:GAP-GFP negative (Figs 3A,D, 4C, 5A and Fig. S10A). At 5 dpf, secondary neuromasts were also *cldnb*:LY-EGFP negative and they partially or totally lacked AP expression (Figs 2A, 4A, 5B and Fig. S10B). When we treated the embryos with 100 µM GSH, we observed a partial rescue of the secondary neuromast phenotypes. In particular, we observed the recovery of *atoh1a*:dTOM and *tnks1bp1*:EGFP expression in secondary LII.1 neuromasts at 4 dpf (Fig. S10A, white arrowheads). Although at 5 dpf the secondary neuromasts also showed *cldnb*:LY-EGFP expression (Fig. S10A, yellow arrowheads), we never observed recovery of *pou4f3*:GAP-GFP expression, suggesting that the antioxidant treatment may not be sufficient to induce full maturation of the hair cells in secondary neuromasts. To perform a more quantitative analysis of the rescue induced by GSH treatment in the embryos and to clearly distinguish between secondary and IC neuromasts, we repeated the same analysis using two different doses of GSH (100 and 200 µM) and then performed AP staining at 4 and 5 dpf. At 4 dpf ∼58% (7/12) of the untreated *ak2^hg14^* embryos had no AP expression in secondary neuromasts (Fig. S10B). However, the 100 µM and 200 µM GSH treatments led to a decrease in this percentage to 47% (8/17) and 33% (6/18), respectively (Fig. 8D). At 5 dpf, we did not observe any AP signal in secondary neuromasts in untreated embryos (Fig. S10B); however, we observed a partial rescue of AP activity in 3/14 (21%) and 4/11 (36%) embryos treated with 100 µM and 200 µM GSH, respectively (Fig. 8D).

Taken together, these data indicate that, as previously observed for hematopoietic phenotypes, the administration of antioxidant treatment with GSH can partially reduce the sensorineural defects observed in an *ak2*-deficient zebrafish model of RD; confirming that similar treatment strategies could potentially be useful in ameliorating non-hematopoietic defects in RD patients.

## DISCUSSION

RD is a rare and severe immunodeficiency disorder also characterized by the presence of sensorineural deafness. In the present study, we took advantage of two different zebrafish models of the disease to extend our knowledge of the physiopathology of RD hearing loss, demonstrating that *ak2* is expressed in vertebrate sensory organs and that it has a crucial role in zebrafish sensory system development. The phenotypes observed in our models of *ak2* deficiency could explain the mechanism that leads to the sensorineural deafness of RD patients. Ak2 deficiency, through increased oxidative stress and cell death, reduces the total number of mature hair cells in the inner ear of mutant animals and it also impairs their regenerative ability. Moreover, it specifically affects the formation of primary and secondary neuromasts of the PLL. Finally, we provided evidence that, as previously observed for hematopoietic defects, ameliorating oxidative stress with antioxidant treatment partially rescues the sensorineural phenotypes observed in the mutant larvae. However, the lower range of doses required to reduce the oxidative stress and to restore the expression of hematopoietic stem cell and lymphoid markers in *ak2* null embryos suggest that hematopoietic cells seem to be more sensitive to the oxidative stress than cells from the sensory system (and thus easier to rescue with antioxidants alone). This idea seems to be supported by the fact that we never observed sensorineural defects in the hypomorphic *ak2^hg16^* animals, although they present the same severe hematological phenotypes as observed in *ak2*-deficient embryos (Rissone et al., 2015).

It is interesting to note that the antioxidant treatments presented a different level of efficacy on the two different parts of the zebrafish sensory system. In the inner ear of *ak2*-deficient mutants the treatment was able to partially rescue the number of mature hair cells (*pou4f*3-GFP^+^) in the cristae and to almost fully restore their correct number in the macula. However, the effect antioxidants had on secondary neuromasts of the PLL was limited to the induction of some of the molecular markers of early neuromasts (*atoh1a*:dTOM and *tnks1bp1*:EGFP, *cldnb*:LY-GFP, and partially AP) and we never observed the formation of more mature *pou4f3*:GAP-GFP*^+^* hair cells. Although there are many potential reasons for this observation, one possible explanation may be that it reflects a major difference in the expression profile of hair and supporting cells between primary and secondary neuromasts, with secondary neuromasts relying more heavily on *ak2* as opposed to other adenylate kinases encoded in the genome. Alternatively, *ak2* may also play distinctive roles during the different stages of neuromast maturation. In particular, our data seem to suggest that during earlier stages of neuromast development, as observed in secondary neuromasts, *ak2* may be involved in maintaining a correct transcriptional profile of the cells, possibly via metabolic feedback. Eventually, during the later phases of neuromast development, the role of *ak2* could be more directly linked to cell survival for both the hair cells and the surrounding supporting cells, as suggested by the increased level of apoptotic cells in the inner ear and primary neuromasts. Notably, altered transcriptional profiles have been previously observed in two different *in vitro* models of RD (Oshima et al., 2018; Six et al., 2015) and, more importantly, it has been shown *in vitro* and in zebrafish that a general reduction of ATP production decreases canonical Wnt/β-catenin signaling, which could result in the observed defects to the IC NMs in *ak2*-deficient embryos (Costa et al., 2019). Another possible scenario is that some pluripotent or multipotent stem cell populations are sensitive to *ak2* deficiencies and are incapable of sustained self-renewal, eventually showing stem cell ‘exhaustion’. In this case, another open question concerns the effects of *ak2* deficiency on specific, non-sensory support cell populations, which represent the pool of progenitors for new hair cells during the regenerative process, and whether they are involved in the pathophysiology of both the zebrafish mutants and the human disease. Further investigations will be required to test these hypotheses.

A variety of mutations have been isolated in RD patients, ranging from missense and nonsense mutations affecting single amino acid positions or *AK2* pre-mRNA splicing, to a range of deletions (Al-Mousa et al., 2016; Al-Zahrani et al., 2013; Guilcher et al., 2017; Heimall et al., 2017; Hoenig et al., 2017, 2018; Lagresle-Peyrou et al., 2009; Pannicke et al., 2009). Although the relative impact of the different genomic mutations has not been systematically evaluated, in most cases the mutations appear to induce a strong reduction of the AK2 protein level, without significantly affecting the mRNA levels (Henderson et al., 2013; Hoenig et al., 2018; Lagresle-Peyrou et al., 2009; Pannicke et al., 2009). A surprising finding with our RD models was that *ak2^hg16^* hypomorphic mutants were able to reach adulthood despite the severe hematological defects observed. This hematopoietic phenotype was shared with the *ak2^hg14^* and *ak2^hg15^* alleles, which were lethal, leading us to believe that the mortality in the stronger alleles was not linked to the initial hematopoietic defects (Rissone et al., 2015). WISH analysis showed that *ak2^hg16^* mutants presented normal levels of *ak2* mRNA expression (although protein levels were not determined), whereas frame-shift mutations in exon 1 in the *ak2^hg14^* and *ak2^hg15^* alleles triggered nonsense-mediated decay of *ak2* mRNA (Rissone et al., 2015). The lack of defects affecting the sensory systems during both larval and adult phases in *ak2^hg16^* hypomorphic mutants despite a strong hematopoietic phenotype strongly suggests that, at least in zebrafish, the hematopoietic lineages are significantly more sensitive to *ak2* reduction than the sensorineural cells.

Although HSCT can correct the immunological defects, extra-immune manifestations are largely not modified and sometimes may become evident many years after a successful transplant. So far, with only one exception (Al-Zahrani et al., 2013), no other major non-hematological defects other than sensorineural hearing loss have been reported in RD patients (Hoenig et al., 2018) despite seeing several affected tissues in the zebrafish models of the disease. Identifying all the possible compromised tissues in RD patients might be important in the future for the early diagnosis of any subsequent non-immunological manifestation in HSCT-treated patients.

## MATERIALS AND METHODS

### Zebrafish lines, maintenance and allele designation

Zebrafish were maintained and used following protocols approved by the National Human Genome Research Institute Animal Care and Use Committee. All protocols and methods related to animals or animal tissues were approved by animal care and use committee of the National Human Genome Research Institute (G-01-3). Zebrafish handling, breeding and staging were performed as previously described (Kimmel et al., 1995; Westerfield, 2000). To prevent pigmentation, the embryos used for WISH or confocal analysis were cultured in fish water containing 0.003% 1-phenyl-2-thiouera (PTU, Sigma-Aldrich, P7629) from 24 hpf. The following strains were used: TAB5 (LaFave et al., 2014); Tg(*-8.0cldnb*:LY-EGFP) (Haas and Gilmour, 2006) (also indicated in the text as *cldnb*); sqet20Et (Hernández et al., 2007; Parinov et al., 2004); Tg(*tnks1bp1*:EGFP;*atoh1a*:dTOM) (Behra et al., 2012); Tg(*pou4f3*:GAP-GFP) (Xiao et al., 2005) (also designated in the text as *pou4f3*); and the Sorbs2b:mRFP1 insertional transgenic line (Clark et al., 2011) in Tg(*pou4f3*:GAP-GFP) background Tg(*pou4f3*:GAP-GFP; mn0002Gt) (designated in the text as *pou4f3:sorbs2b*).

For allele designation, we followed the general rules of zebrafish nomenclature for designating mutant allele names, as described in The Zebrafish Information Network (https://wiki.zfin.org/display/general/ZFIN+Zebrafish+Nomenclature+Conventions). At the ages that experiments were performed, sex of the larvae cannot be determined; it is assumed that male and female fish are equally represented in all experiments.

### WISH

WISH analysis was performed as previously described (Rissone et al., 2015; Thisse and Thisse, 2014), with minor modifications. For *ak2* antisense probes, the hybridization mix was supplemented with 5% w/v dextran sulfate (Thisse and Thisse, 2014). The DIG RNA Labeling kit (SP6/T7; Roche) was used for *in vitro* transcription using PCR products cloned into the pCR4-TOPO vector (Life Technologies) or directly from PCR fragments (Thisse and Thisse, 2014). All probes were purified using Spin Post-Reaction Clean-Up Columns (Sigma-Aldrich) before use. The following DIG-labeled antisense mRNA probes were generated: *eya1*, *atoh1a*, *prdx1-6*, *gpx1a-b*, *gpx4a-b*, *sod1-2*, *gstp1*. See Table S1 for a complete list of primers. All embryos used for WISH were fixed overnight in 4% formaldehyde (PFA)/PBS, rinsed with 0.1% PBS-Tween 20 (PBT), dehydrated in 100% methanol, and stored at −20°C until being processed for WISH. Permeabilization incubation time in 10 µg/ml Proteinase K was optimized to maximize the signal in inner ear or posterior lateral line, with a 1-3 min step depending on the stage (3-5 dpf). Hybridization was performed at 70°C. Stained embryos were stored in 4% PFA until imaging using a ZEISS Axio Zoom V16 stereo microscope. In the images, stained embryos are shown in a lateral or dorsal position with anterior to the left.

### Histology

After WISH staining, 5-day-old embryos were prepared for embedding in paraffin, transferred to paraffin, oriented, sectioned in ∼5 µm transversal sections, and then counterstained using Nuclear Fast Red (Sigma-Aldrich, N3020). Representative pictures were taken using an upright Zeiss Axio Imager D2 microscope (Carl Zeiss) with a Plan-Apochromat 20×/0.75 NA or a dry Plan-Neofluar 40×/0.75 NA objective lens. All images were acquired using an AxioCam HRc full color CCD camera with a 1388×1040 pixel imaging field. Zeiss ZEN blue pro 2011 software package was used for analysis.

### AP staining

AP staining to visualize the PLL neuromasts was performed as previously described (Venero Galanternik et al., 2016). Briefly, after overnight fixation in 4% PFA, the embryos were washed several times in PBT and then transferred to WISH staining buffer. The development of the signal was periodically checked under a stereomicroscope and it was stopped by washing with PBT and finally transferring the embryos to 4% PFA. Stained embryos were stored in 4% PFA until imaging using a Zeiss Axio Zoom V16 stereo microscope. L1 and L2 define primary neuromasts; LII.1 and LII.2 designate the lateral line secondary neuromasts; asterisks indicate IC neuromasts.

### Inner ear dissection and hair cell bundle staining

Inner ear dissections and phalloidin staining of hair cell bundles was performed as previously described (Liang and Burgess, 2009). Briefly, after inner ear fixation in 4% PFA the different compartments of the inner ear (saccule, lagena and utricle) were dissected under a stereomicroscope and then stained with Alexa-labeled phalloidin (Alexa Fluor 488 Phalloidin, 1:1000, Thermo Fisher Scientific) for 30 min at room temperature to visualize hair cell bundles from *ak2^hg16/+^* and *ak2^hg16^* fish. Nuclei were stained with DAPI (Thermo Fisher Scientific, D1306). After the staining, the tissues were washed in PBT three times for 10 min each and then mounted onto a slide with Vectashield Antifade mounting medium with DAPI (Vector Laboratories). Fluorescent signal was analyzed using a Zeiss LSM 880 confocal system (see below). Quantification was performed by selecting representative 2500 µm^2^ areas for every structure (six different areas for utricles, four in the case of saccules and lagenas) and the number of phalloidin-positive bundles in each area was counted. Then the average number was calculated and statistical analysis was performed (see below) (Liang and Burgess, 2009; Zhang et al., 2018).

### Confocal imaging

Confocal images were collected at room temperature using a Zeiss LSM 880 confocal system fitted with an Airyscan module, mounted on an inverted Zeiss Axio Observer Z1 microscope with a Plan-Apochromat 20×/0.75 NA objective lens (Carl Zeiss). Embryos were anesthetized and embedded in 1% low-melting agarose and imaged. All images were acquired in LSM 880 mode using a 32-channel GaAsP-PMT detector. Lasers with excitation wavelengths of 488 nm, 561 nm and 405 nm were used for green, red and DAPI signals, respectively. A range of *z*-slices at optimal intervals was used depending on the zebrafish orientation to capture all desired structures. *z*-sections were collected at defined intervals, and maximum projections were performed on each *z*-stack. All confocal images were of frame size 512×512 or 1024×1024 pixels, scan zoom of 0.6 and line averaged two times. Zeiss ZEN 2.3 (black) software package was used for collection and post-processing of the images. In the images, embryos are shown in a lateral position with anterior to the left. *pou4f3* denotes the Tg(*pou4f3*:GAP-GFP) line labeling the mature hair cells; *atoh1a*:*tnks1bp1* indicates the Tg(*tnks1bp1*:EGFP; *atoh1a*:dTOM) line labeling immature hair cells and supporting cells; *pou4f3*:*sorbs2b* indicates the Tg(*pou4f3*:GAP-GFP; mn0002Gt) double transgenic line labeling mature hair cells and supporting, mantle and interneuromast cells; sqet20Et line labels the mantle cells of rosettes as well as the interneuromast cells.

### Hair cell ablation and regeneration assay

Hair cells were ablated at 3, 4, or 5 dpf by treatment with 10 µM copper(II) sulfate (Sigma-Aldrich, 451657) in 1× Holt's buffer for 1 h (Hernández et al., 2007). After hair cell ablation, the embryos were kept in fresh Holt's buffer for 2 days to allow recovery and hair cell regeneration. Functional hair cells of neuromasts were stained using 2 µM solution of Yo-PRO-1 iodide (Thermo Fisher Scientific, Y3603) in 1× Holt's buffer for 1 h. Stained embryos were washed three times with 1× Holt's buffer and then anesthetized with 0.02% w/v MS-222 (Sigma-Aldrich, A5040) before analysis. The embryos were then transferred to a 96-well plate (one embryo per well) and orientated to a lateral position for counting positive hair cells in the lateral line neuromasts. Positive mature hair cells in L1-L4 lateral line primary neuromasts or in LII.1-LII.2 secondary neuromasts were counted using a Zeiss Axio Observer A1 Inverted Phase Contrast Microscope and then the average number of regenerated hair cells was calculated (Pei et al., 2018, 2016). To confirm the correct ablation of hair cells, a small number of copper-treated embryos were stained using Yo-PRO-1 iodide immediately after the copper treatment.

### TUNEL assay

A TUNEL assay to label apoptotic and dead cells was performed using the ApopTag Red In Situ Apoptosis Detection Kit (Millipore, S7165) following the manufacturer's instructions with minor modifications. Permeabilization incubation time in 10 µg/ml Proteinase K was optimized to maximize the signal in inner ear or PLL as in WISH experiments (1-3 min maximum depending on the stage). In the pictures, embryos are shown in a lateral position with anterior to the left.

### Chemical treatments

Zebrafish embryos were exposed to different doses of l-glutathione reduced (GSH) (Sigma-Aldrich, G4251) from 24 hpf until 5 dpf in E3 embryo medium containing 0.003% PTU. New embryo medium with fresh compound was administered daily until 5 dpf. AG1478 (2-3 µM, Calbiochem, 658552) and 6-BIO (0.2 µM, Sigma-Aldrich, B1686) compounds or the same amount of DMSO (Sigma-Aldrich, D2650) used as control were added to embryo media from 2 to 5 dpf.

### Statistical analysis

Statistical analysis was conducted using two-tailed Student's *t*-test using Excel (Microsoft) or a one-way ANOVA followed by Tukey's post-hoc test (*P*<0.001) to compare groups of fish treated differently using Prism (GraphPad). A difference was considered as significant when the *P*-value was less than 0.05 for Student's *t*-test and less than 0.001 for the Tukey's post-hoc test. Bar graphs show mean and s.d. or s.e.m., depending on the test used. All experiments shown were replicated at least two times.

## Supplementary Material

Supplementary information
