# Supplementary material for: A model for reticular dysgenesis shows impaired sensory organ development and hair cell regeneration linked to cellular stress
Source: Dis Model Mech. 2019 Dec 20;12(12):dmm040170. doi: 10.1242/dmm.040170 (PMC6955229; doi:10.1242/dmm.040170)
Supplement: Supplementary information [file dmm-12-040170-s1.pdf]

Figure S1

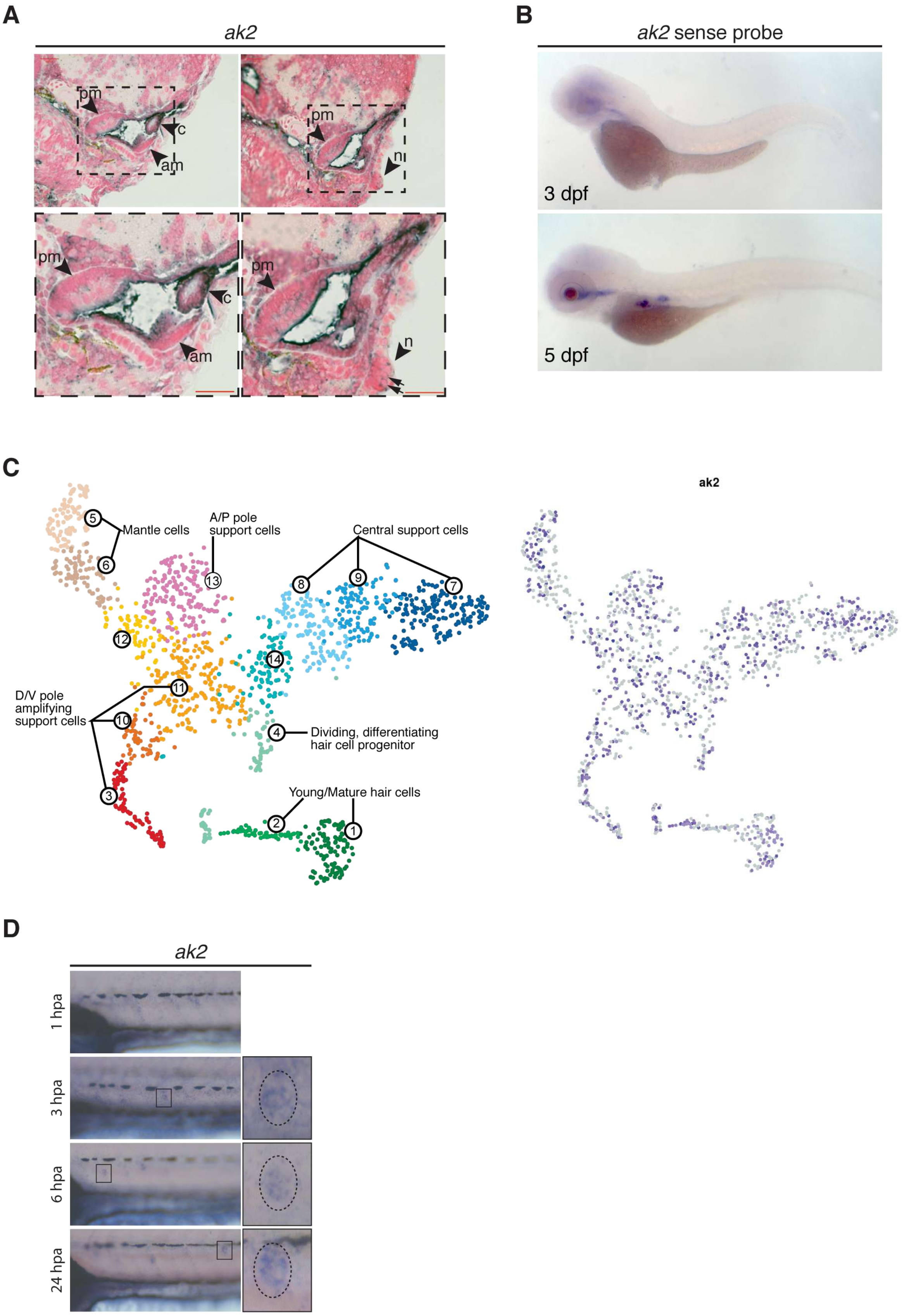

Disease Models & Mechanisms • Supplementary information

Figure S1

***ak2* expression in the otic vesicle and neuromasts in zebrafish larvae.**

(A) Paraffin transverse sections through otic vesicle of 5 dpf wild-type larvae stained with an *ak2* WISH probe (purple). Nuclei are labeled with nuclear fast red. Magnification: 40x. Insets show higher magnifications of the same dashed areas. Black arrows in insets point to *ak2* positive hair cells inside the neuromast structure. Scale bars: 20 µm. c: crista; pm: posterior macula; am: anterior macula; n: neuromast. (B) WISH with an *ak2* sense probe on 3 and 5 dpf wild-type zebrafish larvae. Lateral views with anterior to the left. (C) The t-distributed Stochastic Neighbor Embedding (t-SNE) plots obtained from single-cell RNA-seq analysis of 5 dpf double transgenic Et(Gw57a);Tg(pou4f3) zebrafish larvae expressing GFP reporter in both hair and support cells (from Lush et al. eLife 2019;8:e44431). The panel on the left displays a t-SNE plot highlighting all the different cell clusters; the panel on the right shows the expression of *ak2* gene in the different clusters. (D) Lateral views of the trunk of wild-type larvae stained with *ak2* antisense probe at different hours after HCs ablation with $CuSO_4$. Black boxes indicate the regions shown in the corresponding insets.

Disease Models & Mechanisms • Supplementary information

Figure S2

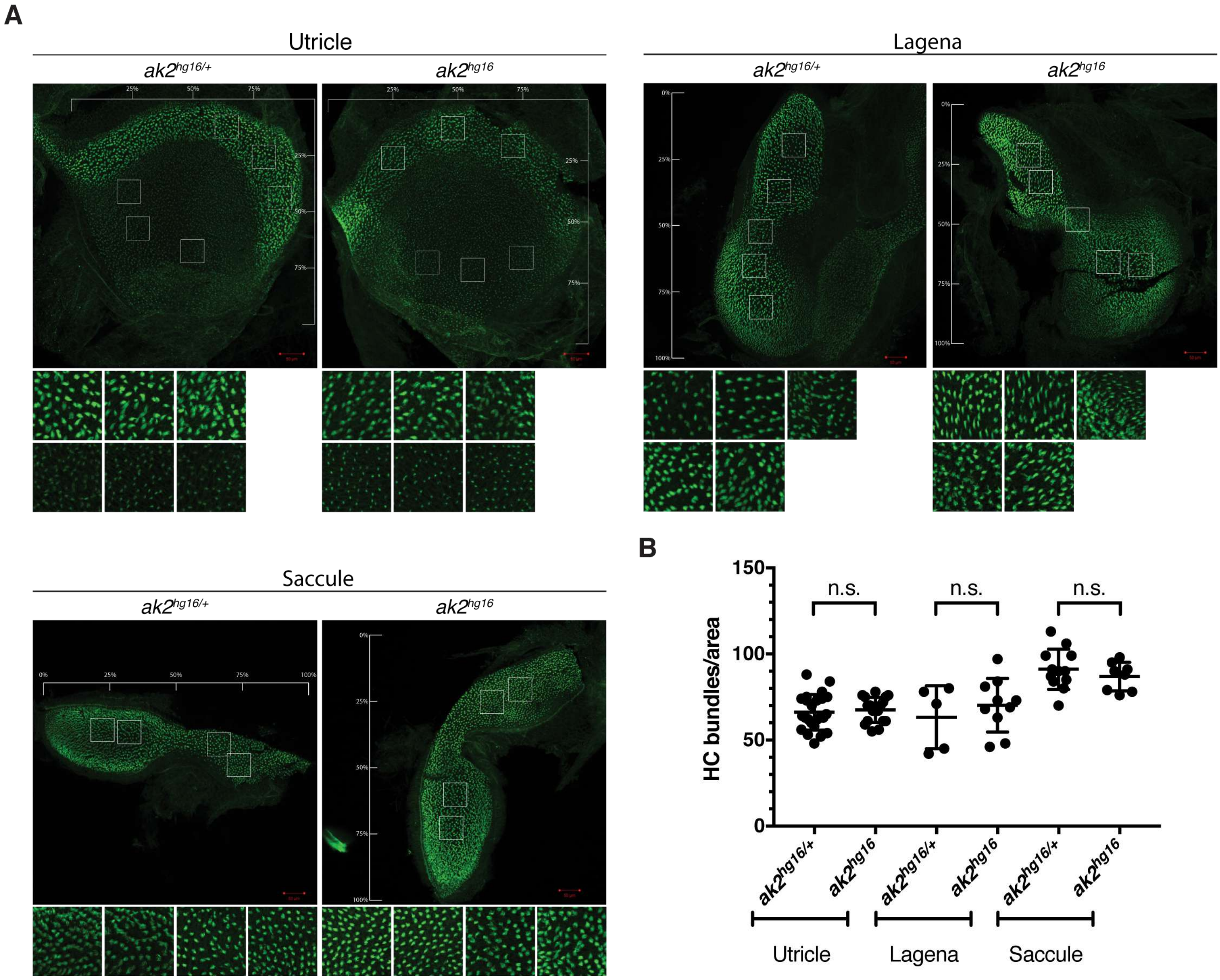

Figure S2

Comparison between hair cell bundles density in utricles, lagenas and saccules of *ak2*$^{hg16/+}$ and *ak2*^{hg16} adult fish.

(A) The hair cell bundles were stained with phalloidin (green) and nuclei with DAPI (blue) (not shown here for clarity). In each structure, hair cell bundle counts were sampled at 2500 μm^2 areas indicated by white boxes and insets below each figure. Scale bars: 50 μm. (B) Quantification and statistical analysis of the hair cell bundle density in *ak2*$^{16/+}$ and *ak2*^{16} homozygous animals of the same age. The error bars indicate standard errors; n.s.: not significant according to a two-tailed unpaired Student's test.

Figure S3

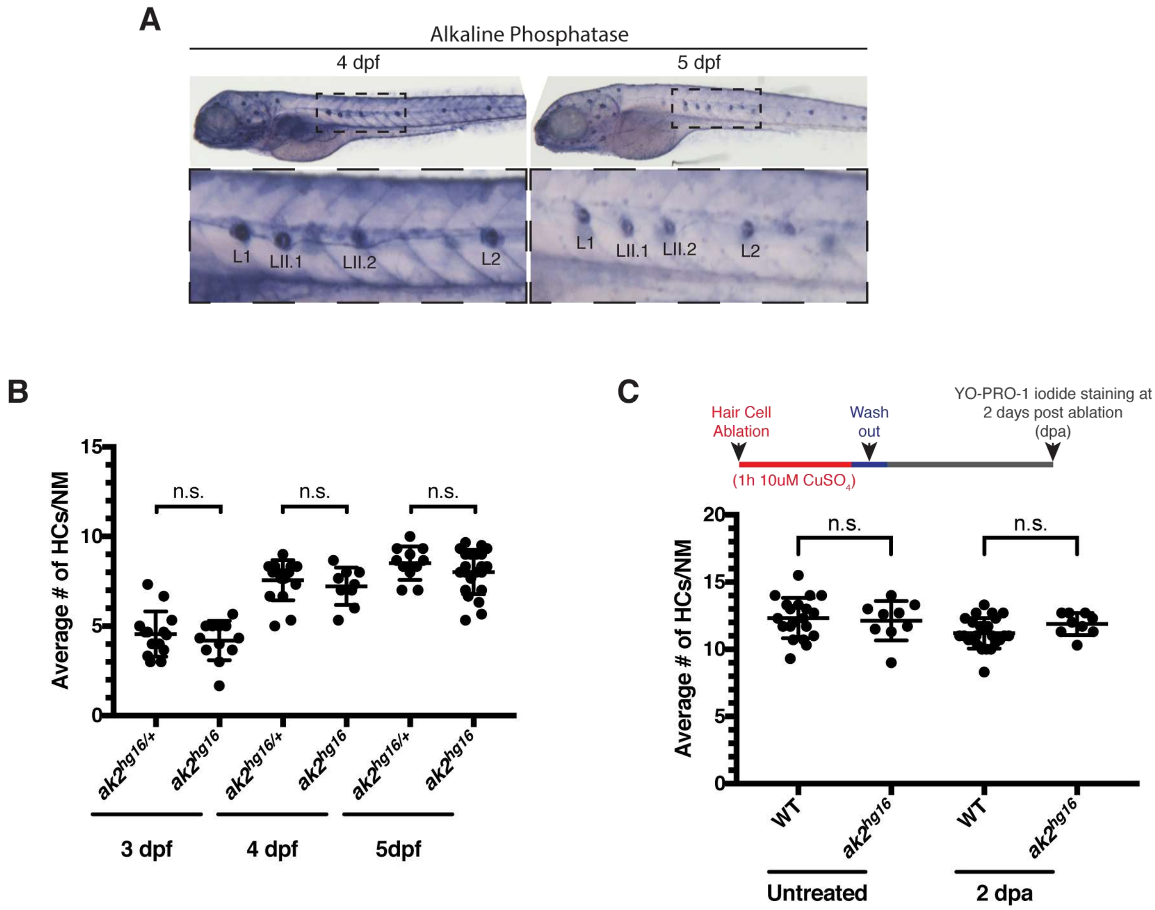

Figure S3

***ak2**^{hg16}* **mutants show no detectable phenotypes in the lateral line neuromasts.**

(A) Alkaline phosphatase staining was performed on 4 and 5 dpf embryos obtained from an incross of *ak2*$^{hg16/+}$ adults. No differences were observed compared to control embryos (see Figure 2A for a refence of AP staining on WT embryos). Lateral views with anterior to the left. Dashed lines indicate insets of trunk regions. (B) Hair cells of lateral line neuromasts were fluorescently labeled with Yo-PRO-1 iodide at different stages to compare the average number of HCs per primary neuromast in *ak2*^{hg16} homozygous and control embryos. (C) Analysis of hair cell regeneration after ablation using $CuSO_4$ treatment followed by Yo-PRO-1 iodide staining in *ak2*^{hg16} embryos and their siblings. The error bars indicate standard errors. n.s. denotes not significant values according to a two-tailed unpaired Student's test.

Disease Models & Mechanisms • Supplementary information

Figure S4

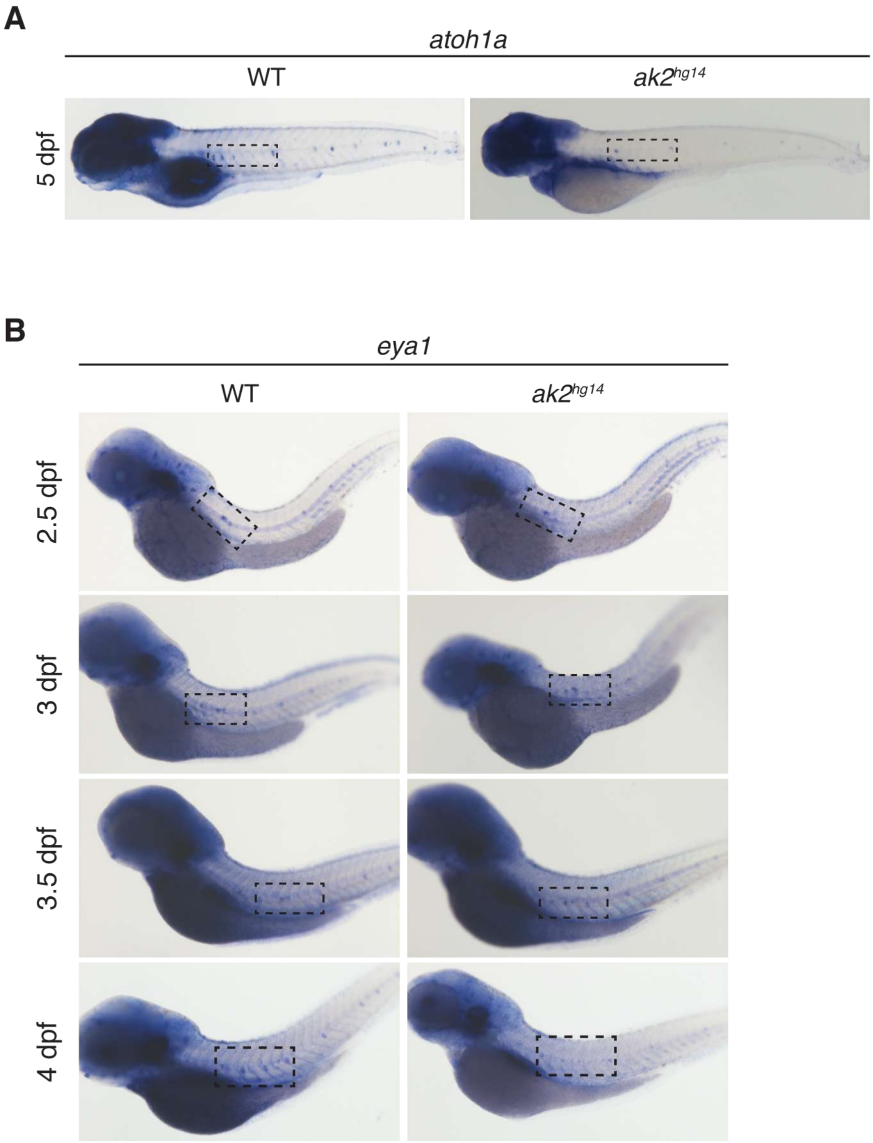

Figure S4

Characterization of *atoh1a* and *eya1* expression in the lateral line of *ak2*^{hg14} mutants.

(A) Expression of the *atoh1a* marker in primary and secondary neuromasts on 5 dpf *ak2*^{hg14} homozygous embryos and their control siblings. Dashed insets indicate the trunk regions shown in Figure 3A. (B) *eya1* gene expression analysis in *ak2*^{hg14} and control embryos at different stages of development (2.5 to 4 dpf). Dashed insets indicate the trunk regions shown in Figure 3C.

Disease Models & Mechanisms • Supplementary information

Figure S5

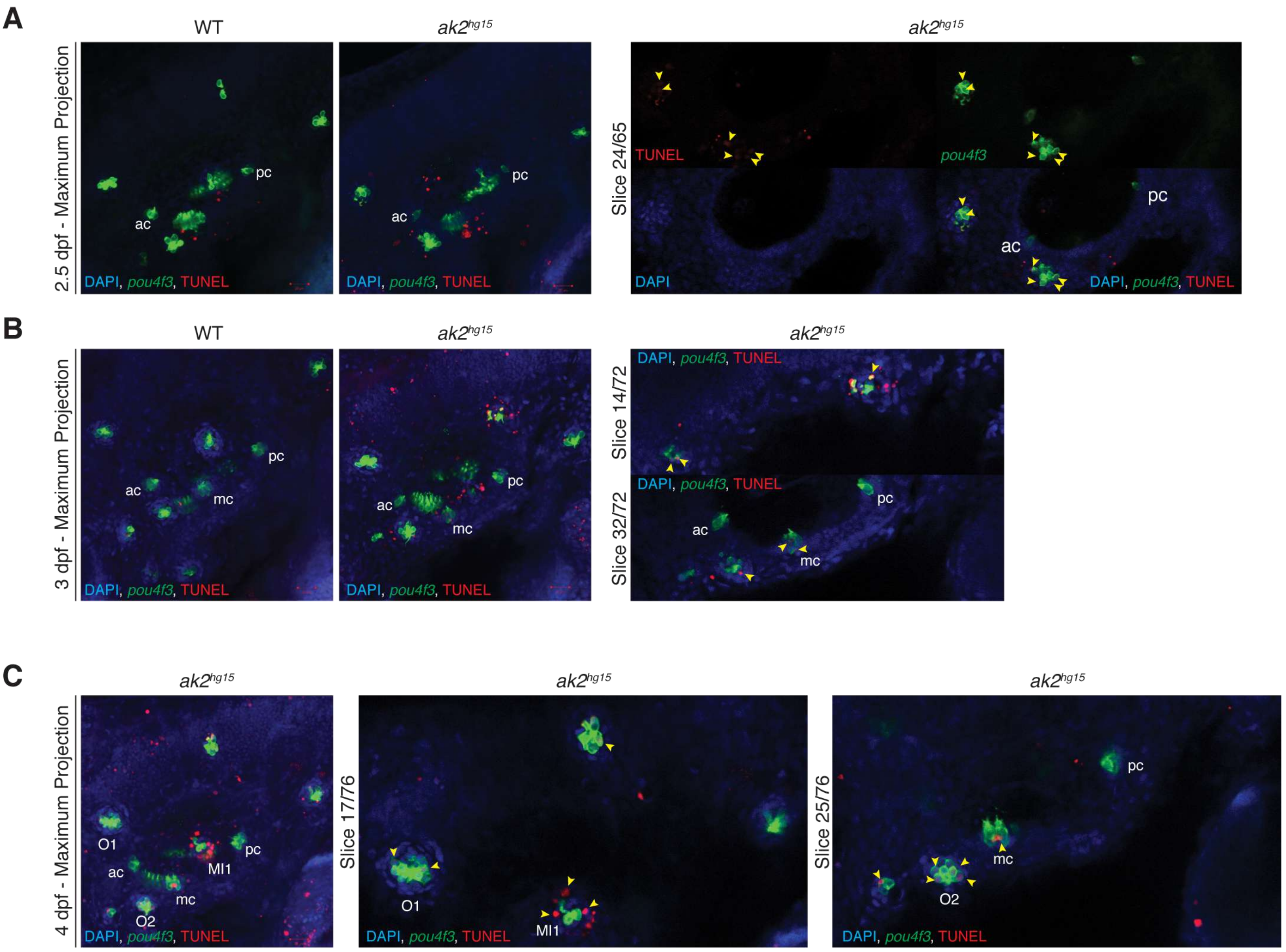

Figure S5

TUNEL staining in the otic vescicles of *ak2*^{hg15} embryos.

(A-C) Maximum projection (left panels) and representative single plane (right panels) confocal analyses at 2.5 (A), 3 (B) and 4 (C) dpf of TUNEL assays (red signal) performed on ak2^{hg15} embryos and their control siblings in the Tg(*pou4f3*:GAP-GFP) background (indicated as *pou4f3*). Yellow arrowheads indicate examples of TUNEL positive cells. Tg(*pou4f3*:GAP-GFP) line labels the mature HCs (green), nuclei are labelled with DAPI (blue). ac: anterior crista; mc: medial crista; pc: posterior crista. Anterior lateral line nomenclature: otic (O1, O2) and middle (MI1) neuromasts. Scale bars: 20 µM.

Figure S6

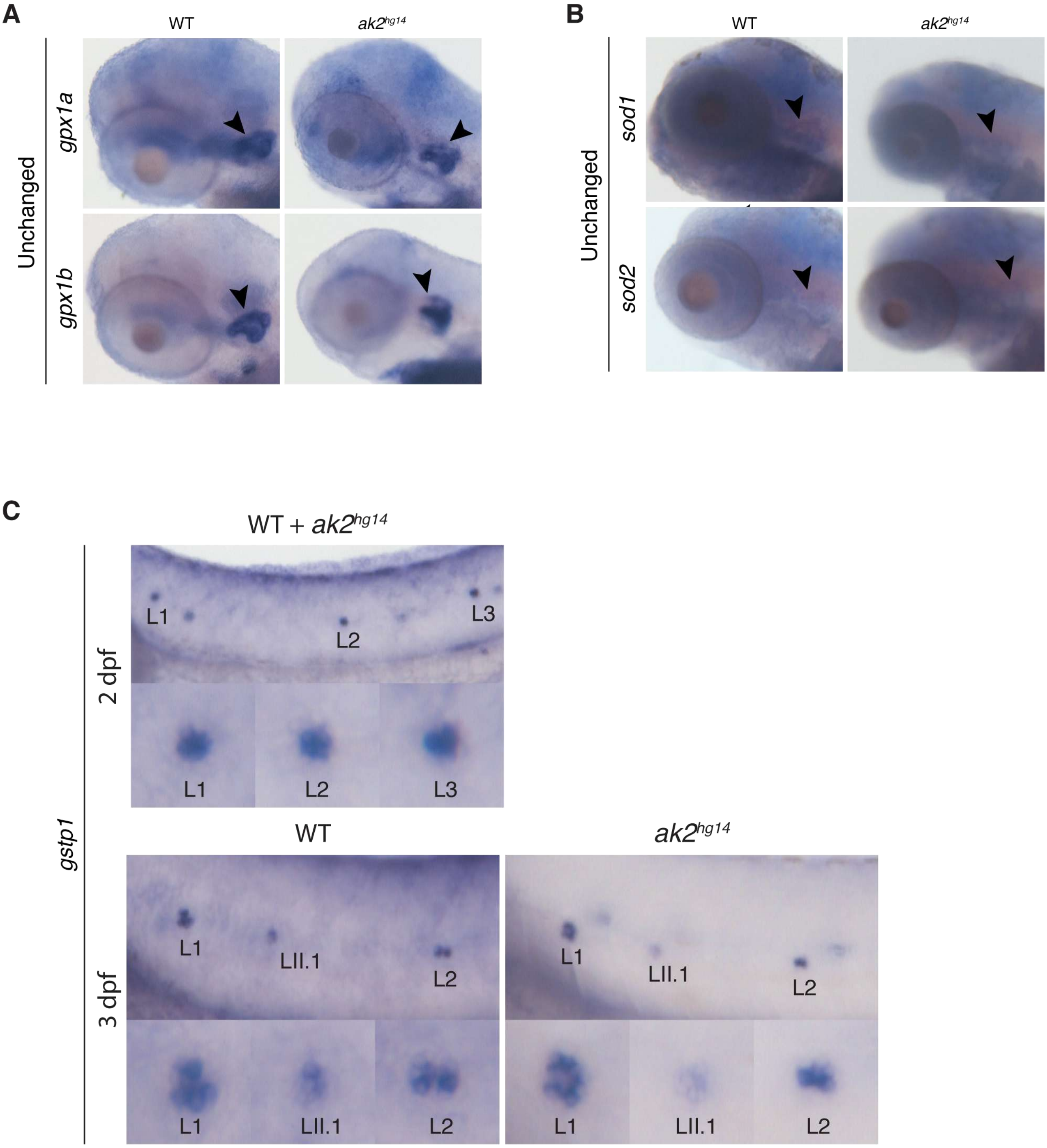

Disease Models & Mechanisms • Supplementary information

Figure S6

WISH analysis of oxidative stress markers in *ak2*^{hg14} embryos.

In situ hybridization analysis of *gpx1a* and *gpx1b* markers at 4 dpf (A), *sod1* and *sod2* markers at 5 dpf (B). The analysis showed no differences in the expression of the markers. (C) WISH analysis of *gstp1* gene expression at 2 and 3 dpf. L1-L3 define primary neuromasts; LII.1 designates the lateral line secondary neuromasts.
Lateral views with anterior to the left. Black arrowheads indicate the otic vescicle. *gpx*: glutathione peroxidase; *sods*: superoxide dismutase; *gstp1*: glutathione S-transferase pi 1.

Figure S7

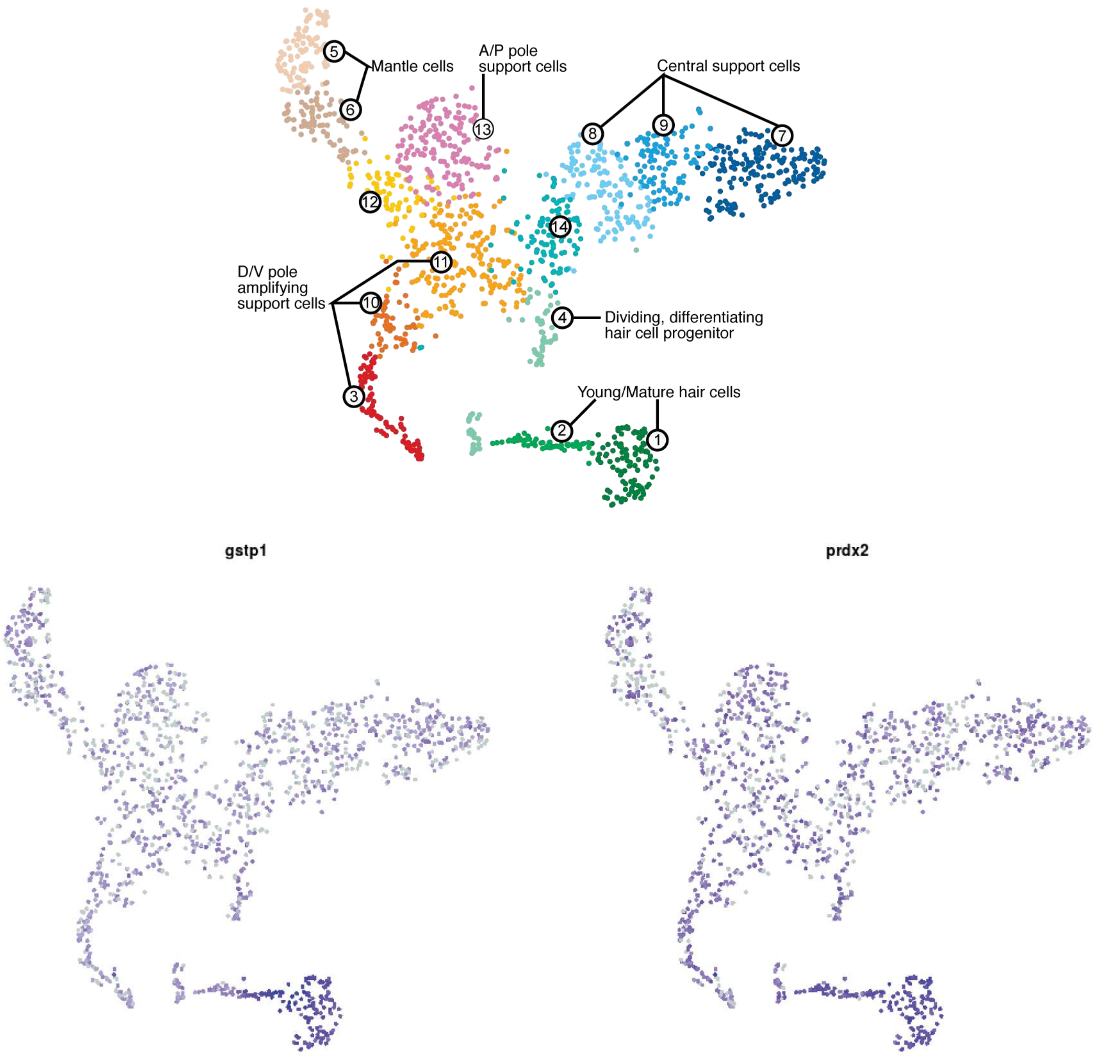

Figure S7

Expression profiles of *gstp1* and *prdx2* genes in neuromasts of 5 dpf zebrafish larvae.

The t-SNE plots obtained from single-cell RNA-seq analysis of 5 dpf double transgenic Et(Gw57a);Tg(pou4f3) zebrafish larvae expressing GFP reporter in both hair and support cells (from Lush et al. eLife 2019;8:e44431). Top panel: t-SNE plot highlighting all the different cell clusters. Bottom panels: t-SNE plots showing the expression of *gstp1* (left) and *prdx2* (right) genes in the different clusters.

Figure S8

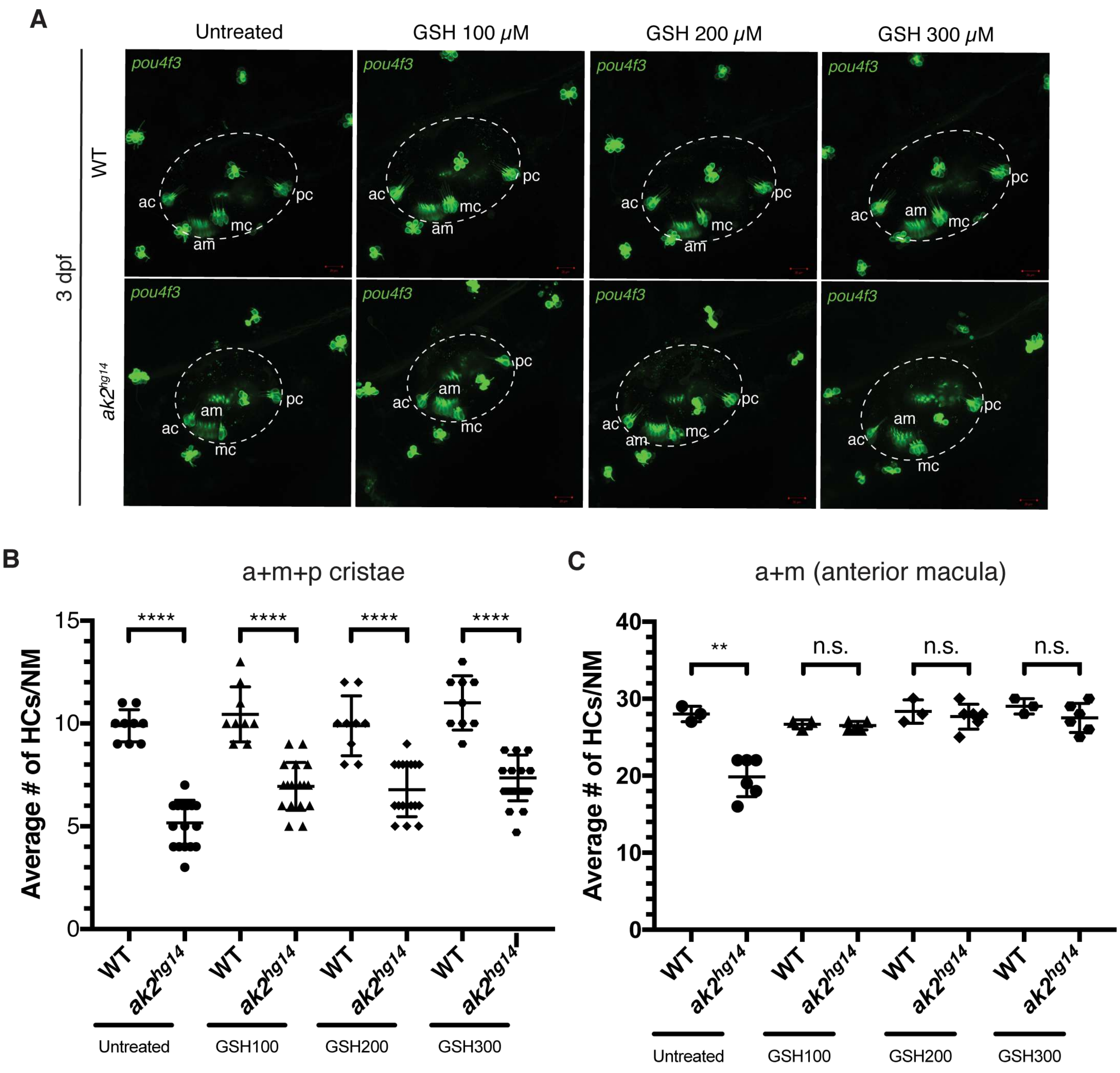

Disease Models & Mechanisms • Supplementary information

Figure S8

Confocal analysis of the inner ear region of *ak2^{hg14}* mutants and their control siblings treated with different concentrations of glutathione until 3 dpf.

Confocal maximum projections of the inner ear region (dashed circles) of 3 dpf Tg(*pou4f3*:GAP-GFP) *ak2^{hg14}* mutants and control siblings, treated with different doses of GSH (100-300 μM). Magnification: 30x, scale bars: 20μm. Lateral views with anterior to the left. *pou4f3* denotes the Tg(*pou4f3*:GAP-GFP) line labelling the mature hair cells (green). ac: anterior crista; mc: medial crista; pc: posterior crista; am: anterior macula. (B-C) Graphs show the comparison of the average number of GFP^+ hair cells in the cristae (a+m+p) (B) and the anterior macula (am) (C) of *ak2^{hg14}* and their control siblings. The error bars indicate standard errors. Asterisks denote statistically significant values according to a two-tailed unpaired Student's test (** = 0.0013; **** = <0.0001). n.s.: not significant.

Disease Models & Mechanisms • Supplementary information

Figure S9

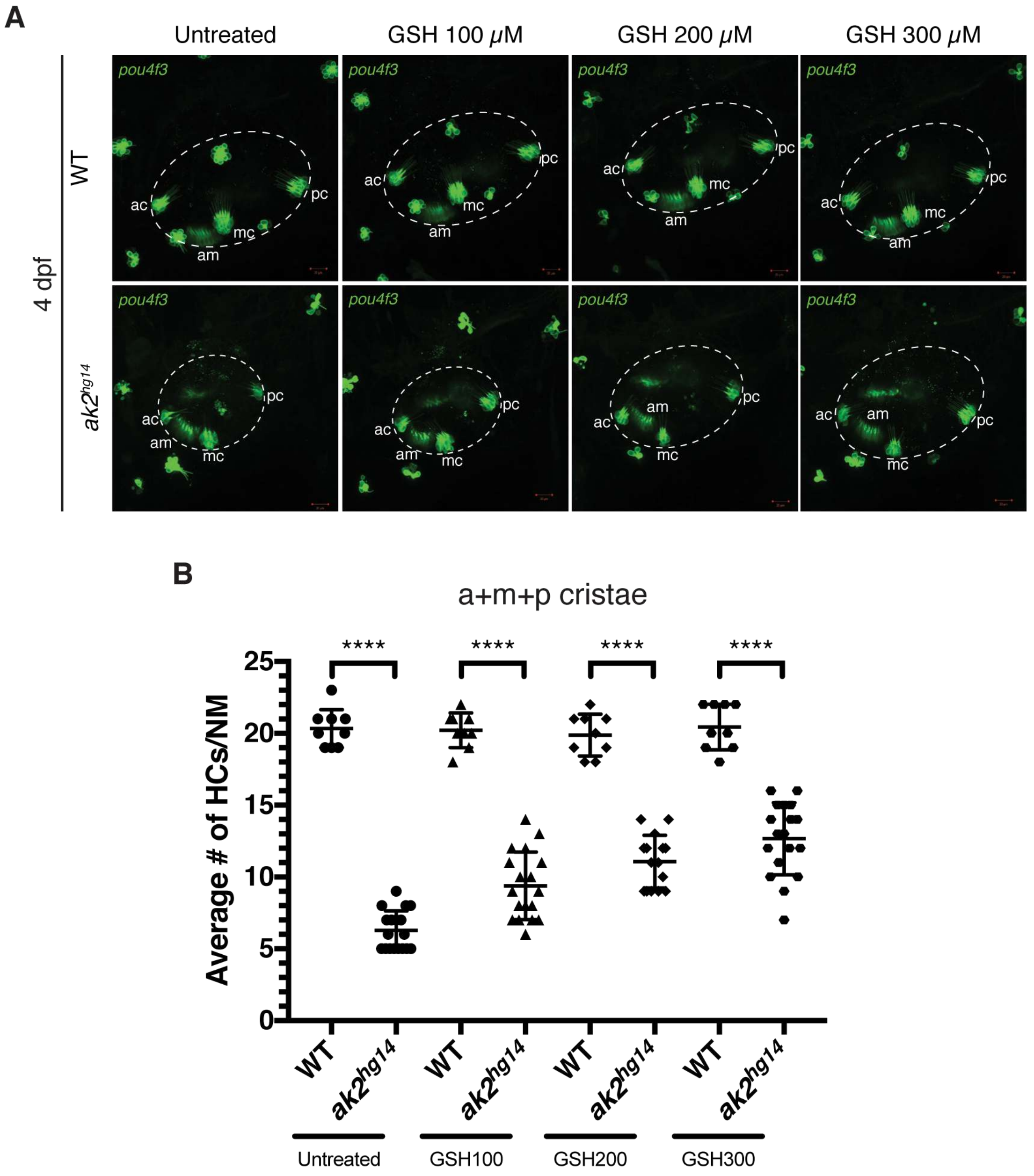

Disease Models & Mechanisms • Supplementary information

Figure S9

Confocal analysis of the inner ear region of *ak2^{hg14}* mutants and their control siblings treated with different concentrations of glutathione until 4 dpf.

(A) Confocal maximum projection of the inner ear region (dashed circles) of 4 dpf Tg(*pou4f3*:GAP-GFP) *ak2*^{hg14} mutants and control siblings, treated with different concentrations of GSH (100-300 µM). Lateral views with anterior to the left. *pou4f3* denotes the Tg(*pou4f3*:GAP-GFP) line labelling the mature hair cells (green). ac: anterior crista; mc: medial crista; pc: posterior crista; am: anterior macula. Magnification: 30x; scale bars: 20µm. (B) Graph showing the comparison of the average number of GFP^{+} hair cells in the cristae (a+m+p) at 4 dpf between *ak2*^{hg14} null and control embryos. The error bars indicate standard errors. Asterisks denote statistically significant values according to a two-tailed unpaired Student's test (**** = <0.0001)

Disease Models & Mechanisms • Supplementary information

Figure S10

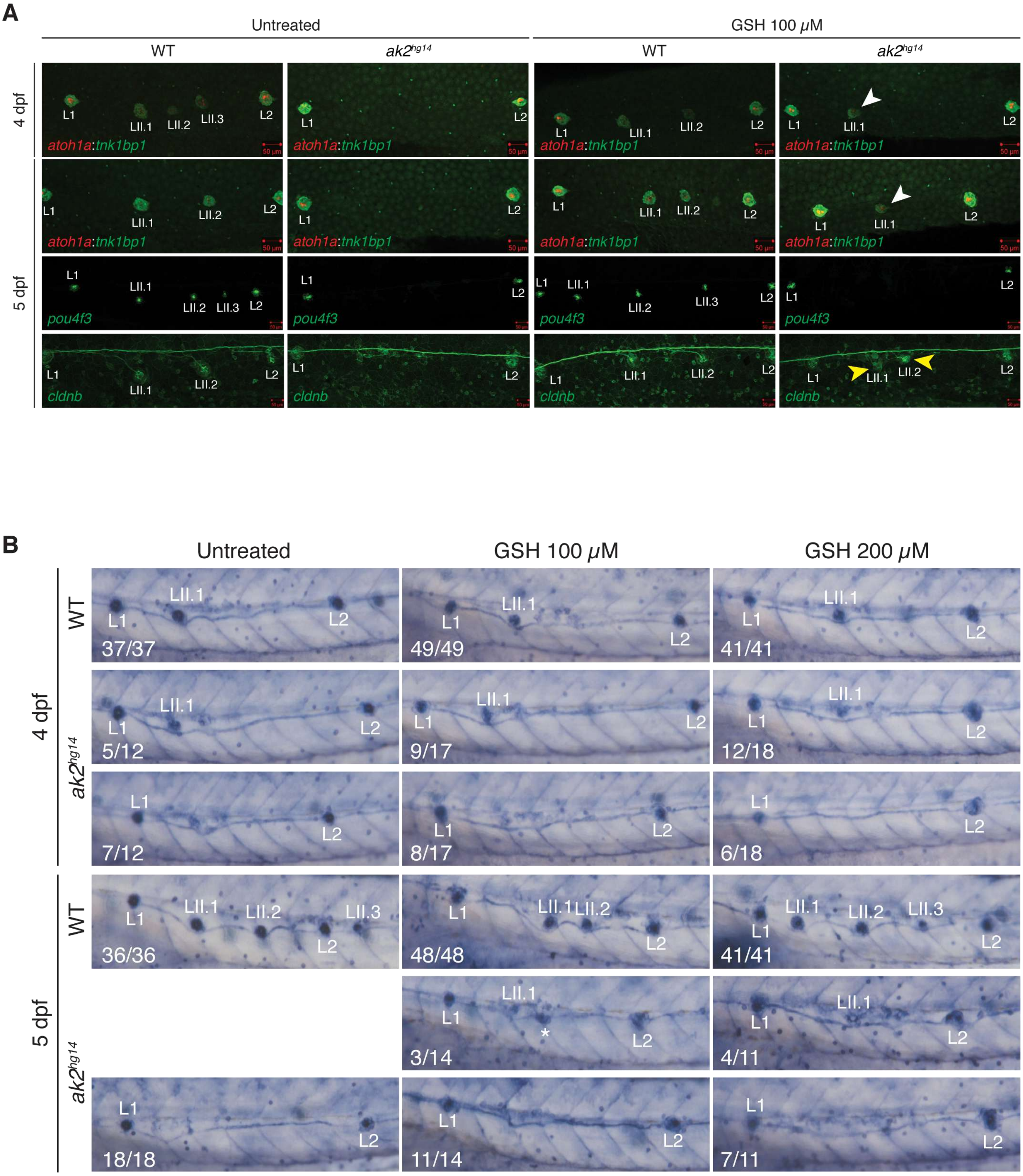

Disease Models & Mechanisms • Supplementary information

Figure S10

Antioxidant treatment partially rescues secondary neuromast defects in *ak2^{hg14}* mutants.

(A) Confocal analysis from 4 to 5 dpf of trunk regions of *ak2^{hg14}* embryos and their siblings in different transgenic background to visualize different components of posterior lateral line.

Untreated embryos are compared to GSH 100 μM treated embryos. *pou4f3* denotes the Tg(*pou4f3*:GAP-GFP) line labelling the mature hair cells (green); *atoh1a*:*tnks1bp1* indicates the Tg(*tnks1bp1*:EGFP; *atoh1a*:dTOM) line labelling immature hair cells (red) and supporting cells (green), respectively. *cldnb* denotes the Tg(*-8.0cldnb*:LY-EGFP) labeling all cells in the deposited neuromasts and the connecting interneuromast cells. White and yellow arrowheads indicate the rescued expression of specific transgenic markers in secondary neuromasts. Scale bars: 50 μm. (B) Alkaline phosphatase staining to visualize the neuromasts in the posterior lateral line of 4 and 5 dpf *ak2^{hg14}* and control embryos untreated or treated with GSH (100 or 200 μM). For each specific phenotype, the number of embryos observed over the total of analyzed embryos are indicated on the bottom left corner.

L1 and L2 label primary neuromasts; LII.1, LII.2 and LII.3 designate the lateral line secondary neuromasts.

Table S1: Primers

Click here to Download Table S1

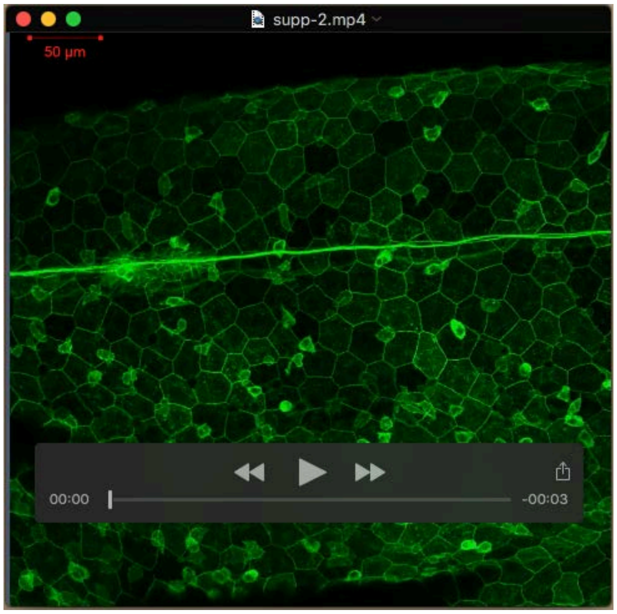

Movie 1.

Time-lapse recording of the migration of primII in an *ak2*^{hg15} Tg(-8.0*cldnb*:LY-EGFP) mutant embryo from ~3.5 to ~4 dpf. Each frame was taken every ~10 min.

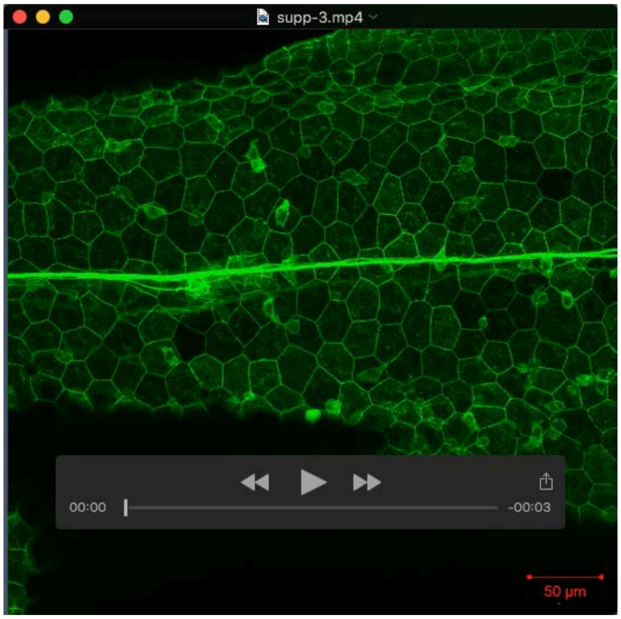

Movie 2.

Time-lapse recording of the migration of primII in a control Tg(-8.0*cldnb*:LY-EGFP) embryo from ~3.5 to ~4 dpf. Each frame was taken every ~10 min.

Disease Models & Mechanisms • Supplementary information
